# Nanopore-Based Neurotransmitter Detection: Advances, Challenges, and Future Perspectives

**DOI:** 10.1021/acsnano.5c04662

**Published:** 2025-06-30

**Authors:** Mostafa Salehirozveh, Parisa Dehghani, Ivan Mijakovic

**Affiliations:** a Systems and Synthetic Biology Division, Department of Life Sciences, Chalmers University of Technology, Gothenburg SE-412 96, Sweden; b James Watt School of Engineering, University of Glasgow, Glasgow G12 8QQ, United Kingdom; c The Novo Nordisk Foundation Center for Biosustainability, Technical University of Denmark, Kongens Lyngby DK-2800, Denmark

**Keywords:** resistive pulse sensing, solid-state nanopore, nanopipette, biosensor, single-molecule detection, neurotransmitter detection, neurodegenerative disease, acetylcholine, dopamine, histamine

## Abstract

Neurotransmitters play a pivotal role in neural communication, synaptic plasticity, and overall brain function. Disruptions in neurotransmitter homeostasis are closely linked to various neurological and neuropsychiatric disorders, including Alzheimer’s disease, Parkinson’s disease, epilepsy, schizophrenia, depression, and amyotrophic lateral sclerosis. This review explores the critical role of neurotransmitters in neurological disorders and highlights recent advances in nanopore-based neurotransmitter detection. Solid-state nanopores (SSNs), with their superior mechanical and chemical durability, have emerged as highly sensitive molecular sensors capable of real-time monitoring of neurotransmitter dynamics. We discuss the integration of SSNs into diagnostic frameworks, emphasizing their potential for early disease detection and personalized therapeutic interventions. Additionally, we examine the complementary role of nanopipettes in neurotransmitter detection, focusing on their high spatial resolution and real-time monitoring capabilities. The review also addresses the challenges and future perspectives of nanopore-based sensing technology, including the need for improved sensitivity, stability, and reproducibility. By integrating insights from neuroscience, bioengineering, and nanotechnology, this review aims to provide a comprehensive overview of how nanopore sensing can revolutionize neurotransmitter analysis and contribute to the development of next-generation diagnostic and therapeutic approaches for neurological diseases.

## Graphical abstract

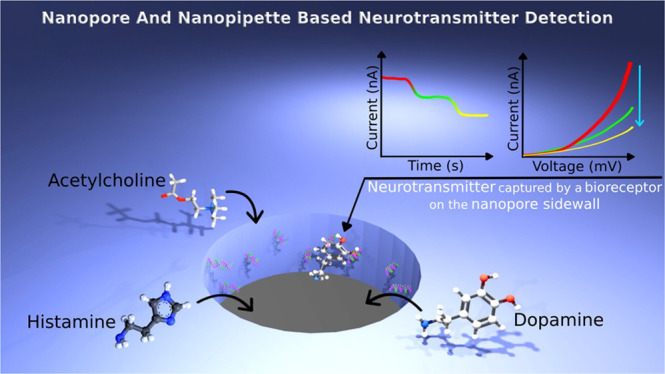

## Introduction

### Significance of Neurotransmitters in Neurological Disorders

Neurotransmitters serve as important chemical regulators for neural communication, synaptic plasticity, and overall brain function.^1^ Maintaining a delicate balance between them is critical for cognitive processes, motor coordination, emotional regulation, and autonomic function.^2^ Disruptions in neurotransmitter homeostasis are closely linked to a variety of neurological and neuropsychiatric conditions, including Alzheimer’s disease (AD), Parkinson’s disease (PD), epilepsy, schizophrenia, depression, and amyotrophic lateral sclerosis (ALS).^3^ understanding of neurotransmitter dynamics in these disorders is essential for advancing early diagnostic strategies, optimizing therapeutic interventions, and developing innovative neurobiological detection methods.^1,3^

Neurotransmitter signaling is coordinated through a complex interplay between excitatory and inhibitory processes, which help maintain neural homeostasis.^4^ Glutamate, a key excitatory neurotransmitter, plays a vital role in synaptic plasticity, learning, and memory; however, excessive glutamate release can lead to excitotoxicity, a major factor in neurodegenerative diseases.^5^ On the other hand, GABA, the brain’s primary inhibitory neurotransmitter, is crucial for neuronal inhibition, and its dysregulation has been linked to disorders such as epilepsy.^5^ Additionally, neurotransmitters like dopamine and serotonin play essential roles in motor control, mood regulation, and cognitive function.^6^ Imbalances in these chemicals contribute to movement disorders like Parkinson’s disease and neuropsychiatric conditions such as depression and schizophrenia.^6^

Early diagnosis and continuous monitoring of neurological disorders are essential for timely intervention, effective disease management, and improved patient outcomes.^7^ Neurotransmitter imbalances serve as key biomarkers for several neurological conditions, including Alzheimer’s disease (AD), Parkinson’s disease (PD), epilepsy, and depression.^8^ Traditional diagnostic methods, such as neuroimaging, cerebrospinal fluid (CSF) analysis, and clinical assessments, often lack the sensitivity needed to detect these disorders in their earliest stages, when treatment interventions may be most effective.^7,9^ Recent advancements in neuroscience and neuroimaging have enabled detailed mapping of neurotransmitter receptor distributions across different brain regions, providing crucial insights into their functional roles and abnormalities associated with neurological disorders.^10^ These studies reveal that such disorders are not merely the result of localized neurotransmitter imbalances but rather arise from widespread disruptions within interconnected neurotransmitter networks.^10^ Additionally, emerging research in epigenetics has shown that neurotransmitter signaling is influenced by both genetic and environmental factors, highlighting the importance of studying epigenetic mechanisms that regulate neurotransmitter biosynthesis, release dynamics, and receptor function.^8^

Continuous monitoring of neurotransmitter levels provides critical insights into disease progression and treatment response.^11^ Conventional methods such as high-performance liquid chromatography (HPLC), mass spectrometry (MS),^12,13^ protein NMR spectroscopy,^14^ enzyme-linked immunosorbent assay (ELISA),^15^ protein immunoprecipitation,^16^ X-ray crystallography,^17^ fluorescence resonance energy transfer (FRET), and electrochemical sensors, have made significant contributions to neuroscience research (Table 3).^18^ However, these techniques often face challenges, including low temporal resolution, complex sample preparation, and the inability to provide real-time measurements in living systems.^11^ Recent advancements in biosensing technologies, particularly nanopore technology and artificial intelligence-integrated platforms, have enabled highly sensitive, label-free detection of neurotransmitters at single-molecule resolution.^19^ These nanopore-based sensors allow for real-time monitoring of neurotransmitter dynamics in both healthy and disease states, opening new possibilities for early disease diagnosis and the development of personalized therapeutic interventions.^11^

This review explores the critical role of neurotransmitters in neurological disorders and highlights recent advances in nanopore-based neurotransmitter detection. By integrating insights from neuroscience, bioengineering, and nanotechnology, we aim to provide a comprehensive overview of how nanopore sensing can revolutionize neurotransmitter analysis and contribute to the development of next-generation diagnostic and therapeutic approaches for neurological diseases.

### Solid-State Nanopore

Nanopores, tiny channels or holes ranging from 1 to 100 nanometres in diameter, are widely used in molecular sensing and filtration applications.^20^ Solid-state nanopores (SSNs) are synthetic nanoscale openings engineered into ultrathin membranes, typically made from materials such as silicon nitride (Si_3_N_4_), graphene, or transition metal dichalcogenides (TMDs).^21^ These artificial nanopores act as highly sensitive molecular sensors, detecting individual biomolecules based on their distinct electrical or ionic signatures.^22^ Unlike biological nanopores, which are protein-based and typically more cost-effective due to their ease of production and self-assembly, they require tightly regulated environmental conditions to maintain structural integrity and functional performance. SSNs, while generally more expensive to fabricate, offer distinct advantages including superior mechanical and chemical stability, longer operational lifespans, and compatibility with large-scale, reproducible manufacturing processes. These attributes make SSNs particularly attractive for robust sensing platforms intended for real-world and long-term applications.^20^ Originally developed for DNA and RNA sequencing, SSN technology has since expanded into a wide range of applications.^23^ These nanopores have demonstrated exceptional effectiveness in detecting proteins, small molecules, and metabolites, making them valuable tools for biomedical diagnostics.^24^ The scalability and portability of devices like the MinION, which are compact and USB-powered, allow for sequencing outside traditional lab environments and accommodate different throughput needs. Furthermore, nanopore technology is cost-effective, offering rapid pathogen detection and other applications at a lower cost. By precisely tuning nanopore size, surface chemistry, and membrane thickness, researchers can optimize SSNs for the selective identification of biomolecules, including neurotransmitters key indicators of brain function and neurological health.^25^

The core mechanism behind SSN-based detection relies on monitoring ionic current fluctuations as molecules pass through the nanopore.^26^ Each neurotransmitter produces a unique current signature based on its size, charge, and interaction with the pore walls, enabling highly specific and label-free identification. Recent advancements in nanopore surface engineering such as chemical functionalization and atomic-layer deposition have further enhanced detection accuracy and selectivity. These innovations allow SSNs to differentiate between structurally similar neurotransmitters like dopamine, serotonin, and glutamate, all of which play key roles in the pathology of neurodegenerative diseases.^25^ The development of nanopore arrays has further expanded the potential of SSNs for high-throughput neurotransmitter screening. Unlike single-nanopore systems, which analyze one molecule at a time, nanopore arrays enable parallelized detection, significantly enhancing analytical efficiency. This advancement is particularly valuable for real-time monitoring of neurotransmitter fluctuations, offering critical insights into synaptic activity and disease progression in conditions such as epilepsy and depression.^27^ With neurodegenerative diseases posing major healthcare challenges, integrating SSNs into diagnostic frameworks presents a noninvasive, highly sensitive approach for early detection and personalized treatment.^28^ Despite these advantages, SSNs face commercialization challenges due to the complexity and cost of their manufacturing processes.^29^ Unlike biological nanopores, which self-assemble naturally, SSNs require advanced nanofabrication techniques such as electron beam lithography, ion beam sculpting, or dielectric breakdown (Figure 1). While effective, these methods remain expensive, limiting the widespread adoption of SSN-based sensors.^28^ The typical manufacturing methods of nanopore arrays are summarized in Table 1. As illustrated in Figure 2, researchers are exploring scalable fabrication techniques, including nanopore array development, which allows for high-throughput molecular analysis and improved detection resolution.^30^

**Figure 1:**
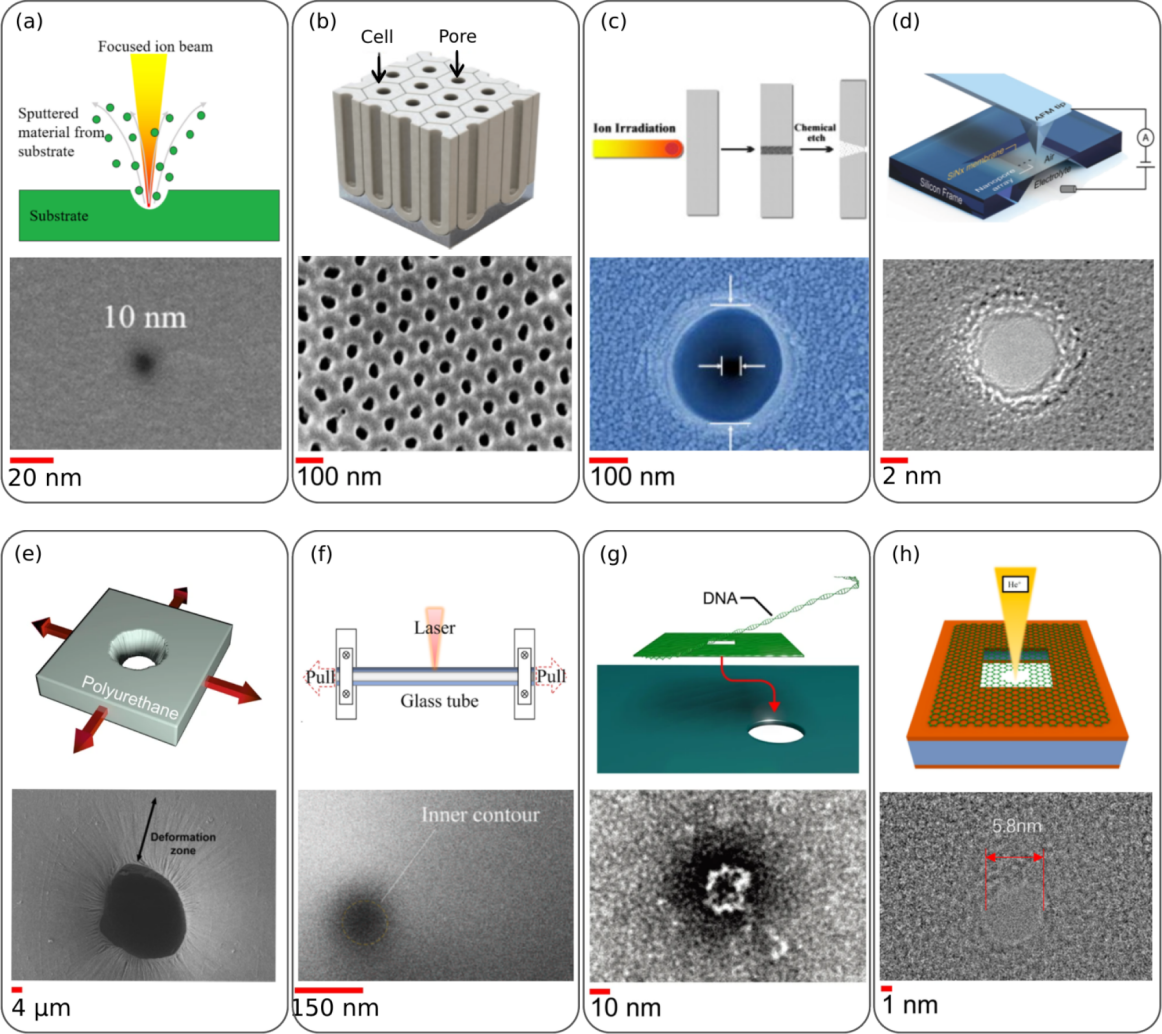
(a) Focused ion beam; reproduced with permissions from ref.^39^ Copyright 2022, Nanotechnology; (b) anodic alumina nanopore. Reprinted with permission under a Creative Commons license, the license CC BY 4.0 from ref.^40^ Copyright 2023, Nanomaterials; (c) track etching, reprinted with permission from ref.^41^ Copyright 2009, Radiation Measurement; (d) AFM tip-controlled local breakdown (TCLB); reprinted with permission from ref.^42^ Copyright 2019, Small method; (e) tunable nanopore, reproduced with permissions from ref.^43^ Copyright 2010, Condensed Matter; (f) glass nanopipette; reprinted with permission from ref.^44^ Copyright 2015, Nanotechnology; (g) DNA origami nanopore, reproduced with permissions from.^45^ Copyright 2012, Nano Letter; and (h) 2D nanopore. Scanning electron micrographs shows the typical nanopore structures fabricated with specific technique. Reprinted with permission under a Creative Commons license, the license CC BY 4.0 from ref.^46^ Copyright 2024, Biosensors.

**Figure 2:**
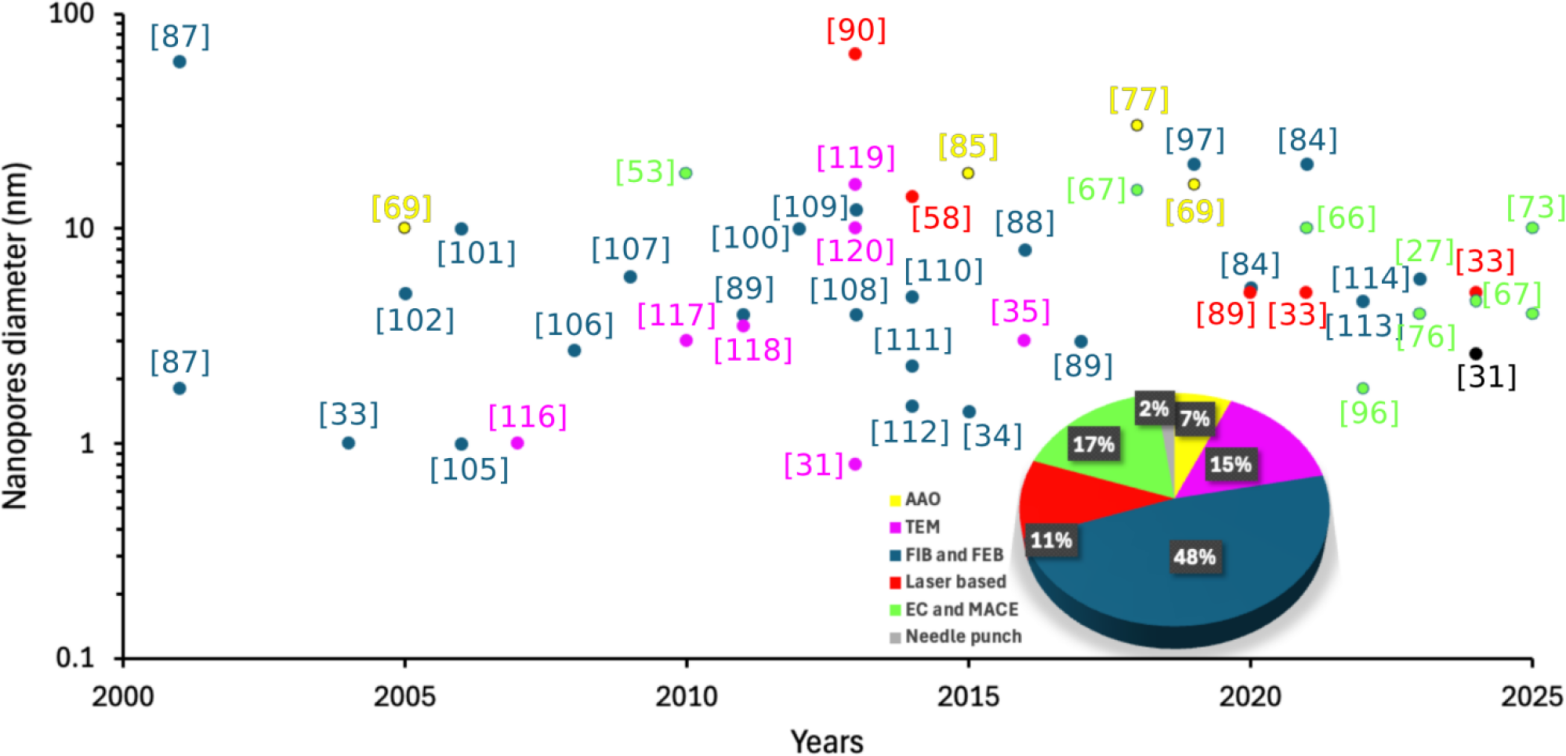
Development of typical solid-state nanopore manufacturing techniques.

**Table 1:** Typical Manufacturing Methods of Nanopore Array^a^

Method	Advantages	Disadvantages	pore size	ref.
Metal-Assisted Chemical Etching	Cost-effective Membrane diversity, Variable thickness	Geometry-dependent	10–300 nm	^32,53,66–69^
Electrochemical anodization, Anodic, AAO, Plasmonic Photochemistry, and photovoltaic electrochemical etch-stop	Thick-pore compatibile, Nanoto-micro scale. High uniformity	Mask-dependent, Material-limited, thick membrane	4–113 nm	^70–78^
Electrodeposition using nanobubbles	Scalable fabrication	Low uniformity	1–160 nm	^79–82^
High-energy beam	Real-time fabrication	Low array yield, Substrate damage risk	0–5 nm	^83–85^
CVD, PVD, ALD	Versatile applications, Fast large-area patterning.	Thick substrate effect, Geometry distortion, Size control challenge	2–20 nm	^76,86–88^
Direct thermal heating	Parallel shrinking, High-efficiency potential	Thick-overdiameter rule	3–20 nm	^89^
Laser etching	Automated NLDA, Fast alkaline etching	Optical condition-dependent, Complex setup	2–5 nm	^33,58,90–92^
Chemical etching	High throughput for thick films	Prepattern required, Slow etching	13–48 nm	^93–96^
Ion and electron beam lithography,And HIM	Precise geometry control, Real-time monitoring, Uniform distribution	Time-intensive, High-cost equipment, Substrate damage risk	0.3–280 nm	^34,46,83,85,87,89,97–113^
TEM and Hybrid fabrication	Customized nanopore, Optimized process, High-quality arrays.	Complex process design, Complex process	80–150 nm	^114–121^
Needle Punching	Low Cost, Simple, Size tunability, High stability, Hybrid fabrication, Scalability.	Material-limited, Complex fabrication, Damage risk, Scaling limitation, Chemical etching	1000 nm	^31^

a Aluminum Oxide: AAO, Chemical Vapor Deposition: CVD, Physical Vapor Deposition: PVD, Atomic Layer Deposition: ALD, nanopore laser drilling algorithm: NLDA, Helium Ion Microscopy: HIM, Transmission Electron Microscopy: TEM.

The use of focused ion beam (FIB) and focused electron beam (FEB) techniques has accounted for approximately 50% of solid-state nanopore fabrications over the past 25 years. Following this, electrochemical (EC) etching and metal-assisted chemical etching (MACE) each account for 17%, with transmission electron microscopy (TEM) at 15%, ranking as the second and third most used solid-state nanopore fabrication methods. As illustrated in Figure 2, Since 2013, laser drilling systems have gained attention in the field, with their usage significantly increased since 2020, particularly for drilling smaller pores. Recently, Rui Liu et al. introduced the needle-punching process with a current feedback system for cost-effective and convenient fabrication of polymer micro/nanopores, with applications in nanofluidic sensing.^31^ Notably, since 2021, researchers have begun integrating machine learning,^32^ feedback control systems,^33^ and automated pore-edge analysis^34^ into these techniques to ensure more reliable, accurate, and reproducible nanopores with consistent size and shape.

### Nanopipettes as a Complementary Technology

Nanopipettes are ultrasmall pipettes with nanometre-scale openings, used in electrochemical, biological, and analytical applications. Nanopipettes are typically fabricated using materials such as silicon nitride (SiNx), silicon oxide (SiO_2_), aluminum oxide (Al_2_O_3_), polyethylene terephthalate (PET) glass, and polyimide membranes, which provide a structurally stable framework to facilitate ion transport.^35^ These nanopipettes, also referred to as nanochannels or nanopores, exhibit biomimetic properties, enabling selective recognition of various target molecules similar to biological ion channels.^36^ Due to their high spatial resolution, minimal invasiveness, and real-time monitoring capabilities, they are widely utilized in single-cell analysis, biosensing, and ion transport studies. While nanopipettes share similarities with nanopores in detecting biomolecules through ionic current changes, they also have distinct advantages and limitations.^37^ In 2022 a review paper explores the use of nanopipettes as sensors, electrodes, and probes.

Like SSNs, nanopipettes detect biomolecules by measuring ionic current modulations as molecules enter and exit the nanopipette tip. This label-free, high-sensitivity technique makes nanopipettes particularly useful for biological and clinical diagnostics.^38^ In a study by Rui Jia et al., researchers focused on single-entity detection using nanopipettes, highlighting their ability to study biomolecular interactions in confined environments.

Their findings demonstrated that by controlling the electric field and fluid flow, nanopipettes can selectively capture and analyze molecules at the single-cell level, making them valuable tools for live-cell studies and intracellular monitoring.^47^ Another review paper examined the evolution of nanopipettes for chemical and bioanalytical applications, emphasizing their role in nanolitre-volume sampling, intracellular probing, and controlled fluid transport. Their ability to precisely regulate sample volumes makes them highly effective for pharmaceutical testing and molecular diagnostics. This capability further enhances their potential in drug development, personalized medicine, and high-precision molecular analysis.^48^

Resistive pulse sensors with nanoscale pores consist of a single, well-defined nanopore embedded within an insulating membrane that separates two electrolyte-filled compartments, each equipped with an electrode (Figure 3a). Nanopore sensing operates by measuring ionic current changes across the pore when a voltage is applied (Figure 3b). As analytes translocate through the nanopore, they displace ions, generating resistive pulse signals that allow for the quantification of molecular passage. Since the nanopore typically represents the highest resistance point in the system, these translocation events induce detectable modulations in the ionic current (Figure 3b, c).^49^ The capture volume in SSNs refers to the spatial region surrounding the pore where charged molecules are influenced by the electric field. Molecule translocation efficiency is governed by the nanopore’s diameter (d) and membrane thickness (L), with smaller diameters and thinner membranes improving spatial resolution for precise molecular detection. Additionally, when an electrolyte buffer carrying poorly conductive or insulated particles flows through the nanopore, particle translocation temporarily increases pore resistance, leading to characteristic ionic current pulses (Figure 3d).^50^ In 2022, Durdane Yilmaz et al. simulated RPS for three nanopore geometries: conical (Figure 3e), cigar-shaped (Figure 3f), and hourglass-shaped (Figure 3g). The study found that particle charge response increases with size. In the conical pore, high particle charge values produced biphasic signals, whereas the cigar-shaped pore exhibited biphasic signals across all charge values for large particles. The cigar pore had the highest charge sensitivity for large and medium particles, making it ideal for charge analysis but less reliable for size discrimination, as charged particles appeared oversized. As particle size decreased, the hourglass pore showed increased sensitivity to charge variations. Moreover, pulse magnitude amplification was highest in the cigar pore, confirming its superior charge sensitivity.^51^

**Figure 3:**
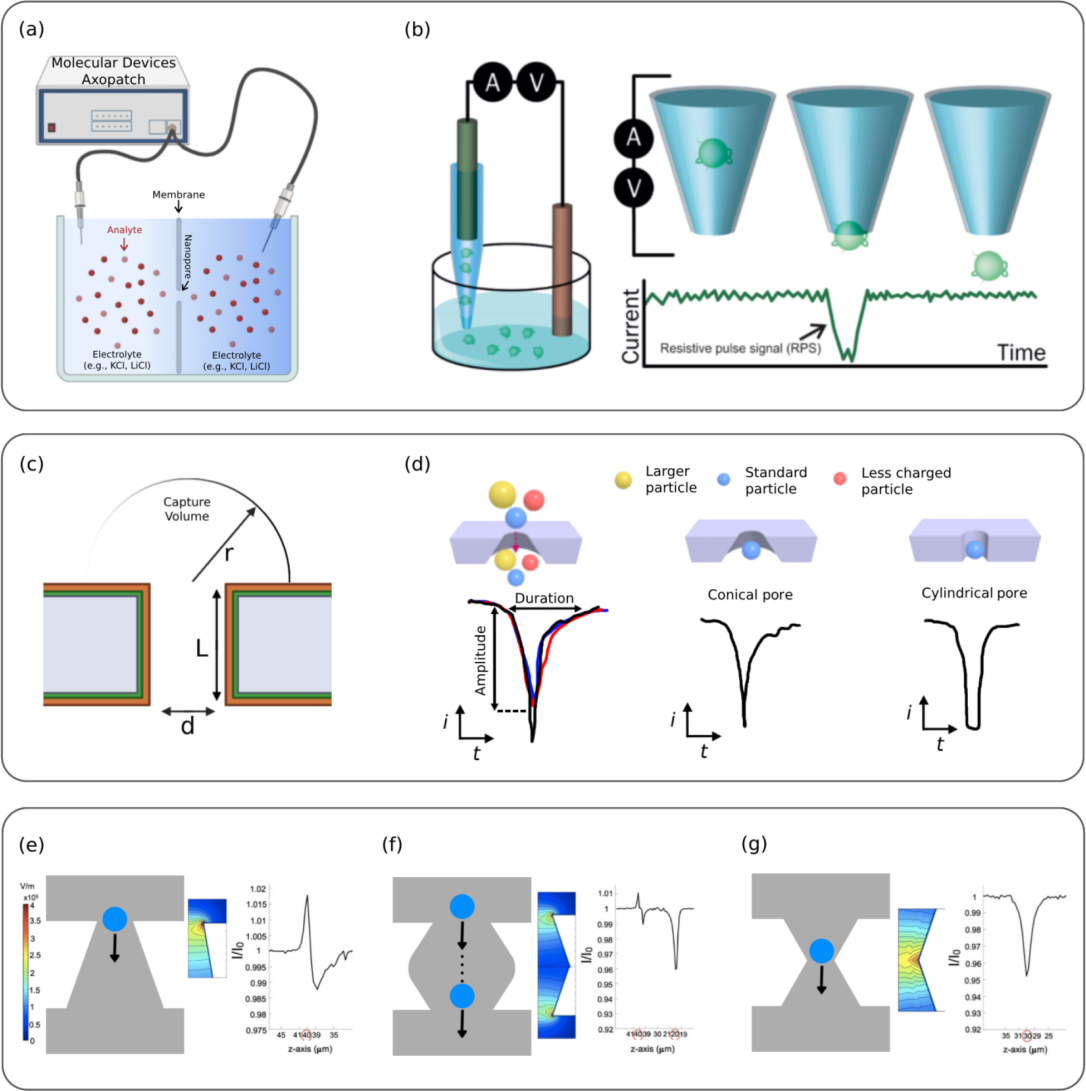
(a) Schematic of a nanopore system showing analyte, ion, and liquid transport. (b) Molecule translocation through a nanopore is facilitated by an applied electric field. As individual molecules pass through the confined pore, they transiently obstruct the flow of ions, leading to discrete disruptions in the ionic current. Reprinted with permission from ref.^65^ Copyright 2020, Biochemical Society. (c) Zoom-in of the pore (diameter d, length L), showing counterions (orange) balancing membrane charge (green). (d) Pulse variations based on particle size, charge, and pore geometry (conical vs cylindrical). Reprinted with permission under a permission under a Creative Commons license, the license CC BY 4.0 from ref.^50^ Copyright 2016, Frontiers Media S.A. **(e–g)** Electric field strength and ionic currents in conical, cigar, and hourglass nanopores for 240 nm particles. adopted with permission from ref.^51^ Copyright 2016, Wiley; John Wiley & Sons.

Nanopore and nanopipette sensing primarily rely on current rectification and RPS techniques.^36,52–54^ In resistive-pulse sensing, two reference electrodes apply a voltage, generating a baseline ionic current (i_0_) through the pore (Figure 4a).

**Figure 4:**
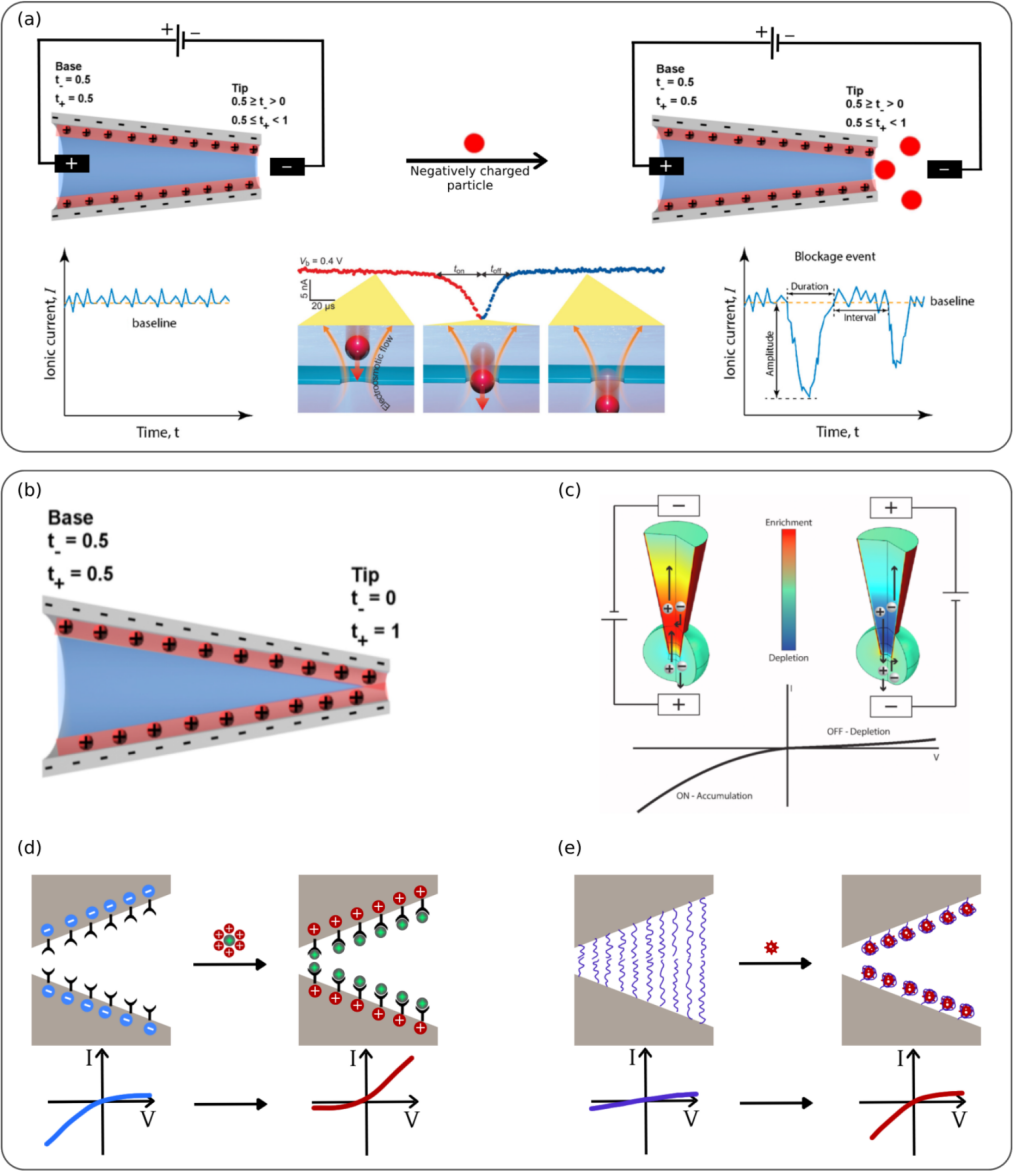
Current Rectification and Resistive-Pulse Sensing in a Nanopipette/Nanopore System. **(a left)** Schematic of a conical nanopore with a tip radius smaller or comparable to the double-layer thickness, where a constant current (i_0_) flows in the absence of analyte. Reprinted with permission under a Creative Commons license, the license CC BY 4.0 from ref.^122^ Copyright 2017, Nanomaterials. **(a middle)** Magnified view of a resistive pulse showing nanoparticle entry into the pore. Insets illustrate the interplay between electrophoretic forces (red arrows) and electroosmotic flow (orange arrows). t_on_ and t_off_ represent capture and escape times. Reprinted with permission under a Creative Commons license, the license CC BY 4.0 from ref.^130^ Copyright 2021, American Chemical Society. **(a right)** Detection of particle translocation, generating current spikes. Dwell time and amplitude provide insights into particle charge and size, while event frequency indicates concentration. Reprinted with permission under a Creative Commons license, the license CC BY 4.0 from ref.^131^ Copyright 2021, American Chemical Society. (b) Schematic of a conical nanopore with a tip radius larger than the double-layer thickness. Red and blue shading represent the double-layer and bulk solutions, respectively. Dimensions are not to scale. Reprinted with permission under a Creative Commons license, the license CC BY 4.0 from ref.^122^ Copyright 2017, Nanomaterials. (c) Ion accumulation and depletion due to nanopore selectivity. Red shading indicates ion concentrations higher than the bulk solution, while blue shading represents lower concentrations. Reprinted with permission under a Creative Commons license, the license CC BY 4.0 from ref.^27^ Copyright 2022, Elsevier. **(d–e)** Target molecule interactions leading to changes in surface charge (d) and nanopore conformation **(e).** Reprinted with permission under a Creative Commons license, the license CC BY 4.0 from ref.^132^ Copyright 2021, Elsevier.

The open-pore conductance (G) in a cylindrical model is determined using eq 1, where s represents solution conductivity, and L denotes membrane thickness.^55,56^ The nanopore or micropore diameter can be derived using eq 2 Based on eq 3, when analyte molecules translocate through the nanopore, they temporarily obstruct the pore opening, causing fluctuations in ionic current. The magnitude of this conductance blockade (ΔG) depends on analyte volume (A), nanopore diameter (d), and effective thickness (h_eff_).^57,58^

$$G=s{\left(\frac{4L}{{\pi d}^{2}}+\frac{1}{d}\right)}^{-1}$$ 1

$$d=\frac{G}{2s}\left(1+\sqrt{1+\frac{16\mathrm{sL}}{\pi G}}\right)$$ 2

$$\Delta G=\frac{\sigma \gamma \Lambda }{{\left(h_{eff}+0.8d\right)}^{2}}S\left(d,a\right)$$ 3

According to DeBlois and Bean,^59^ which is dependent on the nanopore diameter (d) and analyte size (a), can be considered negligible and approach unity^60^ when the analyte diameter (d_m_) is significantly smaller than the nanopore diameter,^61^ and the analyte length (l_m_) remains shorter than the effective nanopore length (h_eff_). Moreover, the orientation of analytes during translocation affects the shape factor (γ), where γ = 1.5 for spherical molecules,^62^ typically leading to a normal distribution of conductance blockade ΔI values.^63^ Consequently, the analysis of analyte translocation signals enables the determination of key parameters such as size, charge, and concentration, along with an estimation of the analyte’s structural shape.

Ion current rectification occurs when the tip radius of a nanopore is comparable to the electrical double-layer thickness at the charged pore walls. When a negatively charged nanopore contacts an electrolyte, a double layer forms, consisting of negative charges on the pore wall and an excess of cations in solution to balance the charge. The thickness of this cation-rich layer, known as the Debye length (λ_d_), depends on electrolyte concentration and decreases with increasing ionic strength, as defined in eq 4.^64^

$$\lambda_{d}=\sqrt{\frac{\varepsilon_{r}\varepsilon_{0}k_{B}T}{\sum_{i}(Z_{i}e{)}^{2}c_{i}}}$$ 4

Where ϵ_r_ is the solution’s dielectric constant, ϵ_0_ the permittivity of free space, *k*_b_ the Boltzmann constant, t the temperature, e the elementary charge, z_i_ the ion valence, and c_i_ the ion concentration. The rectification effect occurs when λ_d_ approaches the pore radius, causing the tip to become cation perm-selective, meaning that only cations contribute to the current flow.

In contrast, at the base of the nanopore, where the double-layer effect is negligible, both cations and anions contribute equally to conduction. This creates an ion current junction, leading to charge depletion at one side and charge accumulation at the other, resembling a semiconductor p–n junction.

While early models assumed rectification required double-layer overlap, experimental studies have shown rectification effects even in nanopores with tip radii exceeding 25–50 nm, which are larger than the Debye length for most electrolyte concentrations. Finite element simulations further validate these rectification behaviors.^122^ Therefore, the solution composition within the nanopore varies along its length. At the base, the solution behaves as a bulk electrolyte, while at the tip, it is composed entirely of a double-layer solution. The double-layer thickness at the base is negligible, ensuring nonpermselective behavior with t_+_ = t_–_ = 0.5 (Figure 4a). Thus, the nanopore acts as an ion current junction, where t_+_ transitions from 0.5 at the base to 1.0 at the tip. This results in charge depletion due to the opposite polarity across the junction, leading to a low current reverse bias state.^122^ The asymmetrical conical ion current junction produces similar effects, where a negatively charged pore cathode at the tip depletes charge carriers under one polarity and accumulates them under the opposite polarity.^123^ As depicted in Figure 4a, double layers overlap in the tip region. However, when the pore radius exceeds the double-layer thickness (Figure 4b), an increased concentration of double-layer cations leads to a scenario where t_+_ > 0.5, resulting in ion current rectification.^124^

In the rectification mechanism, the surface charge of the nanopore or nanopipette is influenced by the charge of the analyte species, leading to ion current rectification effects (Figure 4d and 4e). In nanopore sensing based on ICR, the detection signal originates from the specific interaction between the target analyte and the probe molecule inside the nanopore. This process is affected by several factors, including modifications in the nanopore’s inner surface charge, effective inner diameter, and hydrophilic–hydrophobic surface properties, either individually or simultaneously. Nanopore sensors typically operate in aqueous environments with charged, hydrophilic molecules. Target binding introduces additional charges, altering the nanopore’s inner surface charge (Figure 4d). Analyte binding can induce conformational changes in nanopore molecules, altering diameter and surface charge, which affect ion transport and modulate conductivity. These shifts in the I–V curves (Figure 4e) enable accurate and sensitive analyte quantification. These sensing techniques have been widely applied for the detection and characterization of single nanoparticles, macromolecules, biomolecules, and vesicles, offering high sensitivity and real-time monitoring capabilities.^47,52,125–129^ The comparison between nanopore and nanopipette is presented in Table 2.

**Table 2:** Comparison between Nanopore and Nanopipette

Feature	Nanopore	Nanopipette	ref
Operation’sprinciples	Ionic current modulations as a function of molecules pass through a nanometre-sized pore	Electrochemical sensing via ionic current changes at a nanoscale tip	^38,48^
Applications	DNA sequencing, biomolecule detection, biosensing, disease diagnostics	Single-cell analysis, neurotransmitter detection, intracellular probing, nanofluidic	^37,47^
Single-Molecule Sensitivity	Extremely high (capable of detecting individual nucleotides and proteins)	High, especially with functionalized tips	^142,143^
Spatial Resolution	Moderate (detects molecules as they pass through the pore but lacks precise spatial resolution)	High (nanoscale control over sample positioning, capable of subcellular imaging)	^37,142^
Selectivity	Enhanced through surface modifications (e.g., graphene-coated, protein-functionalized nanopores)	Can be enhanced with functionalization (e.g., aptamers, enzymes, chemically modified nanopipette tips)	^37,143^
Signal Stability	Can suffer from noise and clogging due to prolonged use and accumulation of biomolecules in the pore	Higher due to stable probe positioning and fewer issues with clogging	^47,48^
Real-Time Monitoring	widely used in DNA sequencing and real-time biomolecule detection	particularly in single-cell analysis and electrochemical sensing	^37,38^
Reusability	Often single-use or has limited reusability due to pore clogging and degradation	Can be reused with proper cleaning and recalibration	^37,48^
Compatibility with Live Cells	Less invasive but typically used for extracellular sensing (membrane-bound nanopores)	Suitable for intracellular studies and single cell probing	^37,48^
Detection of Small Molecules	High sensitivity but requires surface modifications for small-molecule differentiation.	Highly sensitive for neurotransmitters and biomolecules (e.g., serotonin, acetylcholine)	^47^
Detection of Large Molecules	Well-suited for DNA, RNA, proteins, and other macromolecules	Effective for proteins, viruses, and macromolecules but may require tip modification	^37,48^
Ease of Fabrication	Well-established in commercial applications (Oxford Nanopore sequencing)	Requires precise nanofabrication techniques but is scalable for laboratory use	^48,143^
Portability	Some solid-state nanopore platforms are portable (MinION for DNA sequencing)	Can be miniaturized for point-of-care applications and lab-on-chip devices	^37,47^
Integration with Microfluidic Systems	Possible, used in advanced lab-on-chip designs for controlled sample handling	Widely integrated into lab-on-chip devices for DNA sequencing and biosensing	^37,143^

### Nanopore and Nanopipette-Based Dopamine Sensor

Dopamine (DA) is an important neurotransmitter involved in most physiological functions, and its abnormal levels are linked to neurological disorders such as schizophrenia and Parkinson’s disease.^25^ Sensitive and precise detection of DA is crucial for clinical diagnostics and biomedical research. Traditional techniques like high-performance liquid chromatography (HPLC), optical, and electrochemical sensors have limitations, including complex sample preparation and interference from other biomolecules (Table 3).^133^ Nanopore-based sensing has emerged as a promising approach due to its real-time monitoring and label-free, high-sensitivity detection.^25^

**Table 3:**
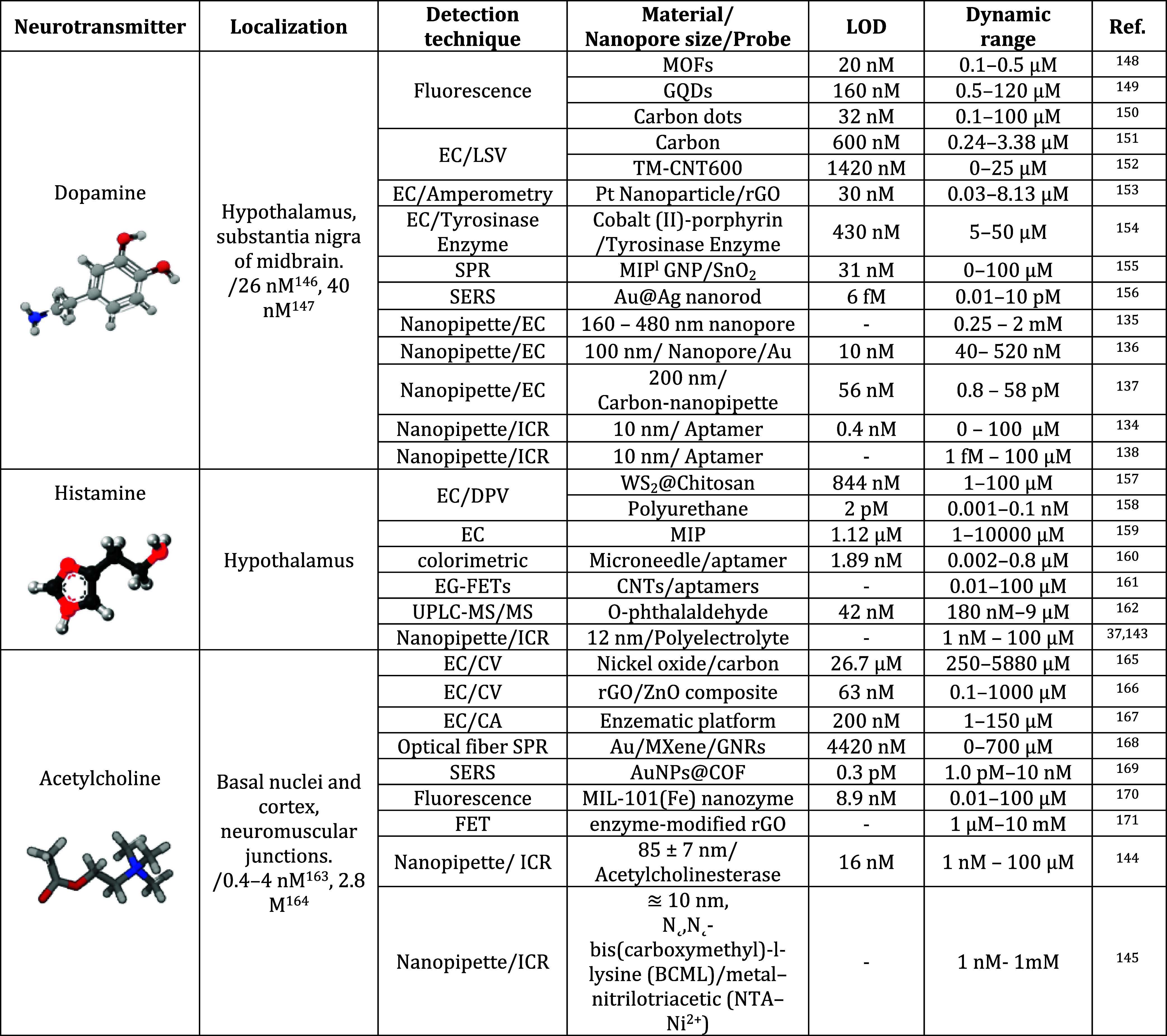
Comparison between Other Techniques and Nanopore and Nanopipette Technologies for Neurotransmitter Detection^a^ ^134,135,136,137,138,139,140,141,142,143,144,145,146,147,148,149,150,151,152,153,154,155,156,157,158,159,160,161,162,163,164,165,166,167,168,169,170,171^

a EC: Electrochemical, LSV: Linear sweep voltammetry, SPR: Surface plasmon resonance, SER: Surface-enhanced Raman spectroscopy, DPV: Differential pulse voltammetry, EG-FET: Electrolyte-gated field-effect transistor, UPLC-MS/MS: Ultraperformance liquid chromatography-tandem mass spectrometry, CA: Chronoamperometry, FET: Field effect transistor MMOF: Metal organic frameworks, GQD: Graphene quantum dots, MIP: Molecular Imprinted Polymer, CNT: Carbon nanotubes, r-GO: Reduced graphene oxide, GNR: Graphene nanoribbons, COF: Covalent organic framework.

Recent advancements in dopamine detection using SSNs have focused on improving selectivity, stability, and detection limits. Several novel strategies have been introduced to enhance the performance of nanopore sensors, including dual-nanopore biosensors, aptamer-functionalized nanopores, and atomic-layer-deposited nanopores. In a recent study presented by Tao Zhao et.al, they introduced a dual-nanopore biosensor designed for both intracellular and extracellular dopamine detection at single-cell level.^134^ In this approach, they used two nanopores to enhance detection selectivity and minimize interference from other biomolecules. The system enables precise, label-free dopamine detection at physiological concentrations.

The dual-nanopore configuration improves signal resolution and reduces background noise, further increasing detection accuracy. Also, functionalizing nanopores with dopamine-specific aptamers has proven to be an effective strategy for improving detection performance.^25^ Here, an aptamer undergoes conformational changes upon binding to DA, resulting in distinct ionic current fluctuations as the complex translocate through the nanopore (Figure 5). The ability to distinguish structurally similar neurotransmitters such as serotonin and norepinephrine further demonstrates the potential of aptamer-functionalized nanopores in multiplex neurotransmitter sensing applications. While this method allows for highly selective and sensitive detection, achieving femtomolar detection limits, and making it suitable for ultralow concentration measurements in biological fluids, its reliance on precise nanopore fabrication and chemical modifications poses challenges in scalability and reproducibility (Table 2). Additionally, the inability to monitor rapid, real-time changes in DA levels due to the high-affinity aptamer design highlights a limitation in capturing dynamic neurotransmitter fluctuations. SSN, fabricated from materials like silicon nitride or graphene, provides greater mechanical stability and tunability in nanopore size. In a study conducted by Michelle L. and et.al, solid-state nanopores were used for DA detection with high specificity, leveraging functionalized surfaces to enhance molecular recognition.^135^ Moreover, machine learning was applied to assist nanopore sensing to enhance DA quantification accurately by analyzing the complex current signals generated during detection.^134^ To improve selectivity in DA detection, chemical modifications of nanopores are another approach. Here, Dan Yang et.al presented the nanopores functionalized with cyclodextrins and aptamers to differentiate dopamine from structurally similar neurotransmitters like norepinephrine and epinephrine.^136^

**Figure 5:**
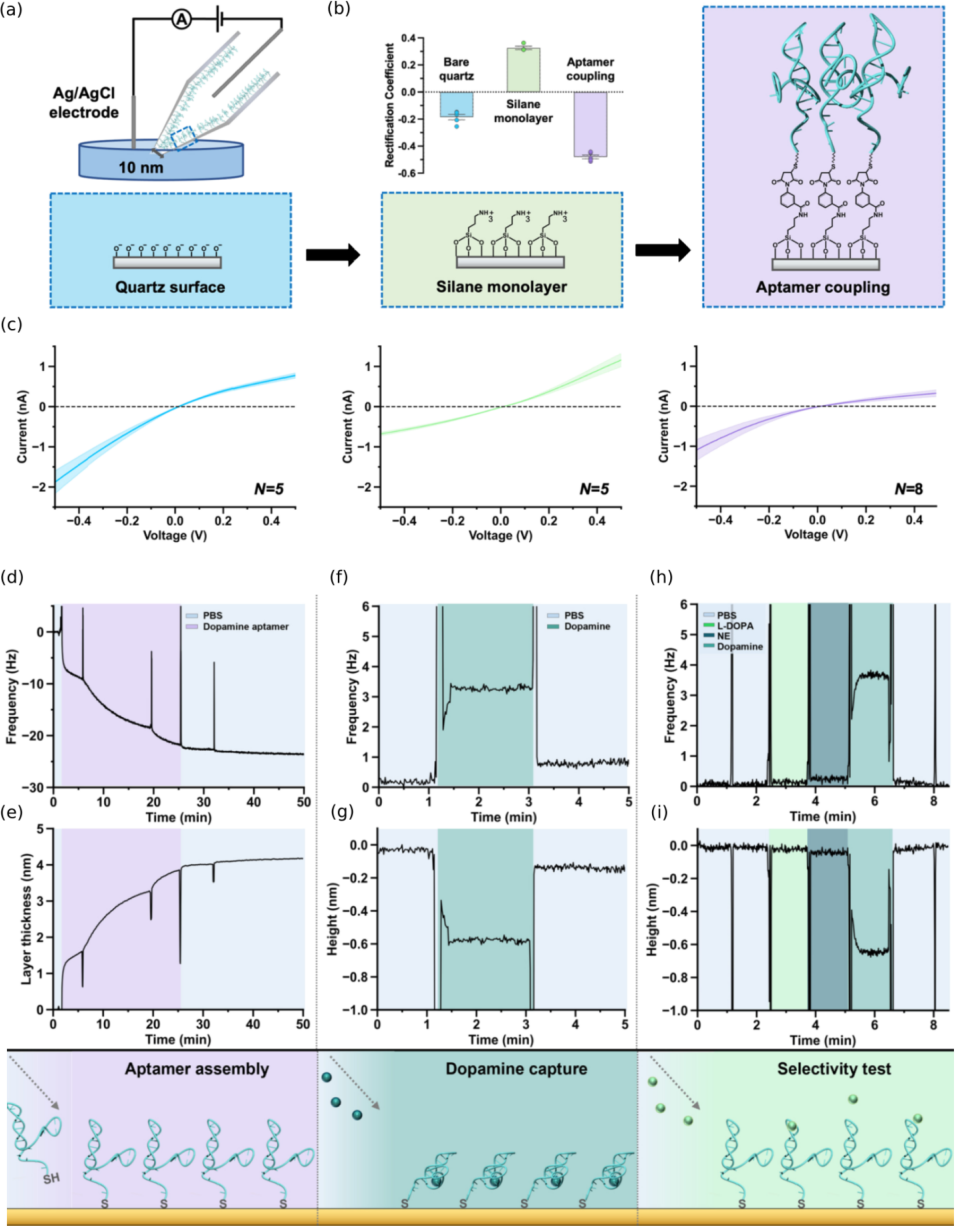
(a) Schematic of surface modification process of nanopipette sensors with aptamer. (**b**) The surface charge was studied after each modification step by calculating the rectification coefficient. (c) Dopamine aptamers, modified through sequential surface chemistry, can be monitored using ion current rectification (ICR), which appears as asymmetric current–voltage curves. Initially, bare quartz nanopipettes (*N* = 5) have negative charges, which turn positive after aminosilane assembly (*N* = 5). Adding negatively charged aptamers increases rectification (*N* = 8). The solid line indicates the average, while the shaded area shows the standard error of the mean. (d) by aptamer functionalization, the frequency was decreased around 23.7 Hz in phosphate buffered saline (PBS). (e) The 4.1 nm thickness of aptamer layer was calculated (f) DA-aptamer interaction led to an increase in frequency to be around 3.3 Hz. (g) which is equal to a 0.6 nm compression in the modified-aptamer layer as a result of DA binding (based on the Sauerbrey equation). (h) In selectivity test, no change was observed following by adding nonspecific molecules such as l-3,4-dihydroxyphenylalanine (L-DOPA) and norepinephrine (NE), **(i)** which did not affect the thickness of modified-aptamer layer. Reprinted with permission under a Creative Commons license, the license CC BY 4.0 from ref.^25^ Copyright 2023, American Chemical Society.

Another promising development involves the use of atomic-layer deposition (ALD) to fabricate highly stable nanopores, improving both structural durability and sensing precision.^133^ ALD-derived HfO_2_ nanopores exhibit superior chemical stability and nanometre-level precision, allowing for highly reproducible dopamine detection in complex biological environments. This approach significantly enhances sensor longevity and robustness, making it suitable for long-term monitoring applications in clinical and research settings. Moreover, the controlled pore size and surface properties of ALD-fabricated nanopores help optimize signal-to-noise ratios, further improving detection resolution. Additionally, Cheng Yang et al. enhanced the sensing capabilities of nanopore-based systems by investigating the influence of surface charge and pore geometry on DA transport and signal resolution.^137^ In their study, they developed cavity carbon-nanopipette electrodes (CNPEs) that exploit a distinct cavity architecture designed to trap DA molecules, thereby increasing local analyte concentration and significantly improving electrochemical detection sensitivity. This strategic design enabled CNPEs to achieve high sensitivity coupled with nanoscale spatial resolution, making them particularly effective for neurochemical sensing applications (Table 3). Despite these advancements, the fabrication process, which relies on chemical vapor deposition (CVD), poses challenges related to precision control and scalability. The requirement for stringent process conditions may hinder large-scale production and practical implementation. Additionally, while the detection limit was notably enhanced, it remains susceptible to baseline system noise. Furthermore, the performance of CNPEs can be compromised by the nonspecific adsorption of biomolecules present in biological tissues, potentially impeding efficient electron transfer at the electrode interface. These limitations underscore the need for further material optimization and surface engineering to ensure consistent performance in complex biological environments. Recent developments in electrochemical nanopore and nanopipette sensors have advanced the detection of DA at nanomolar concentrations through integration with redox-active probes.^138^ A key outcome of this work is the detailed characterization of the nanopipette sensor’s operational mechanisms, particularly its reliance on ion current rectification and aptamer-mediated molecular recognition. These mechanisms contribute to the sensor’s selectivity and sensitivity (Table 3). Importantly, the study demonstrates successful quantification of DA in complex biological matrices, including blood serum and neuronal culture media, signifying a major advancement toward real-world applications in neuroscience research and clinical diagnostics. Despite these promising capabilities such as nanoscale spatial resolution and real-time detection several challenges persist.

The reproducibility of sensor responses in variable ionic environments remains a significant concern, often resulting in performance inconsistencies. Moreover, the fabrication process demands stringent environmental control to ensure the structural and functional stability of the device. Notably, the presence of divalent cations has been shown to markedly influence aptamer behavior, further complicating sensor performance in diverse biological settings. These limitations highlight the critical need for continued optimization of sensor design, surface chemistry, and fabrication protocols to improve reliability, reproducibility, and adaptability across a broader range of physiological conditions.

Recent advances in electrochemical sensing have enabled in vivo DA detection, offering high spatial and temporal resolution in live brain tissue.^139^ Techniques such as nanocavity electrodes and nanoelectrode arrays have been employed for real-time monitoring of dopamine release during neuronal activity.^140^ While nanopore-based sensors have primarily demonstrated success in in vitro and intracellular environments, adapting them for in vivo applications remains an emerging research frontier, with ongoing efforts aimed at improving sensor stability, biocompatibility, and resistance to biofouling.

### Nanopore Technology for Acetylcholine (Ach) Detection

Acetylcholine (ACh) is a key neurotransmitter involved in neuromuscular function, cognitive processes, and autonomic nervous system regulation. Dysregulation of ACh levels is linked to neurodegenerative diseases such as Alzheimer’s disease and myasthenia gravis. Traditional detection methods, including enzymatic assays and electrochemical sensors, often suffer from limitations such as interference, long detection times, and complex sample preparation. Nanopore-based sensing has emerged as a promising technique due to its high sensitivity, real-time detection, and label-free analysis. For ACh detection functionalized nanopores can significantly enhance the specificity and sensitivity of sensing by modifying the pore surface with molecular recognition elements such as enzymes, aptamers, and synthetic receptors. A study by Yamili Toum Terrones et al.^141^ explores the critical role of surface charge modulation and functionalization in enhancing nanopore selectivity for neurotransmitter detection. Their work highlights the electrostatic self-assembly of polyethylenimine (PEI) onto poly­(ethylene terephthalate) solid-state nanopores (PET/PEI SSNs), which significantly improves the differentiation of ionic current signals between ACh and structurally similar neurotransmitters (Figures 6b and 6c). The research introduces a highly sensitive biosensing strategy that incorporates acetylcholinesterase (AChE)-modified nanochannels. This design leverages enzymatic amplification mechanisms to boost detection precision and reliability (Table 3). A pivotal advancement in this approach is the integration of weak polyelectrolytes as dynamic “chemical amplifiers.” These materials adjust the nanochannel’s surface charge in real time, responding to localized pH shifts generated by AChE-catalyzed reactions. This mechanism enables reproducible and sensitive detection of ACh at nanomolar concentrations. The PET/PEI/AChE SSN system operates effectively across both nanomolar and micromolar concentration ranges (Figures 6e and 6f), positioning it as a promising tool for neurochemical sensing (Table 3). Despite these strengths, several challenges limit the broader application of this technology. Sensor stability can be compromised under fluctuating pH and ionic conditions, which directly impact the performance of the polyelectrolyte-based amplification mechanism. Furthermore, the dependence on enzymatic activity introduces variability in detection efficiency, and the precise fabrication required for bullet-shaped nanochannels restricts scalability. Additionally, signal specificity may be affected by interference from nontarget biomolecules in complex biological samples.

**Figure 6:**
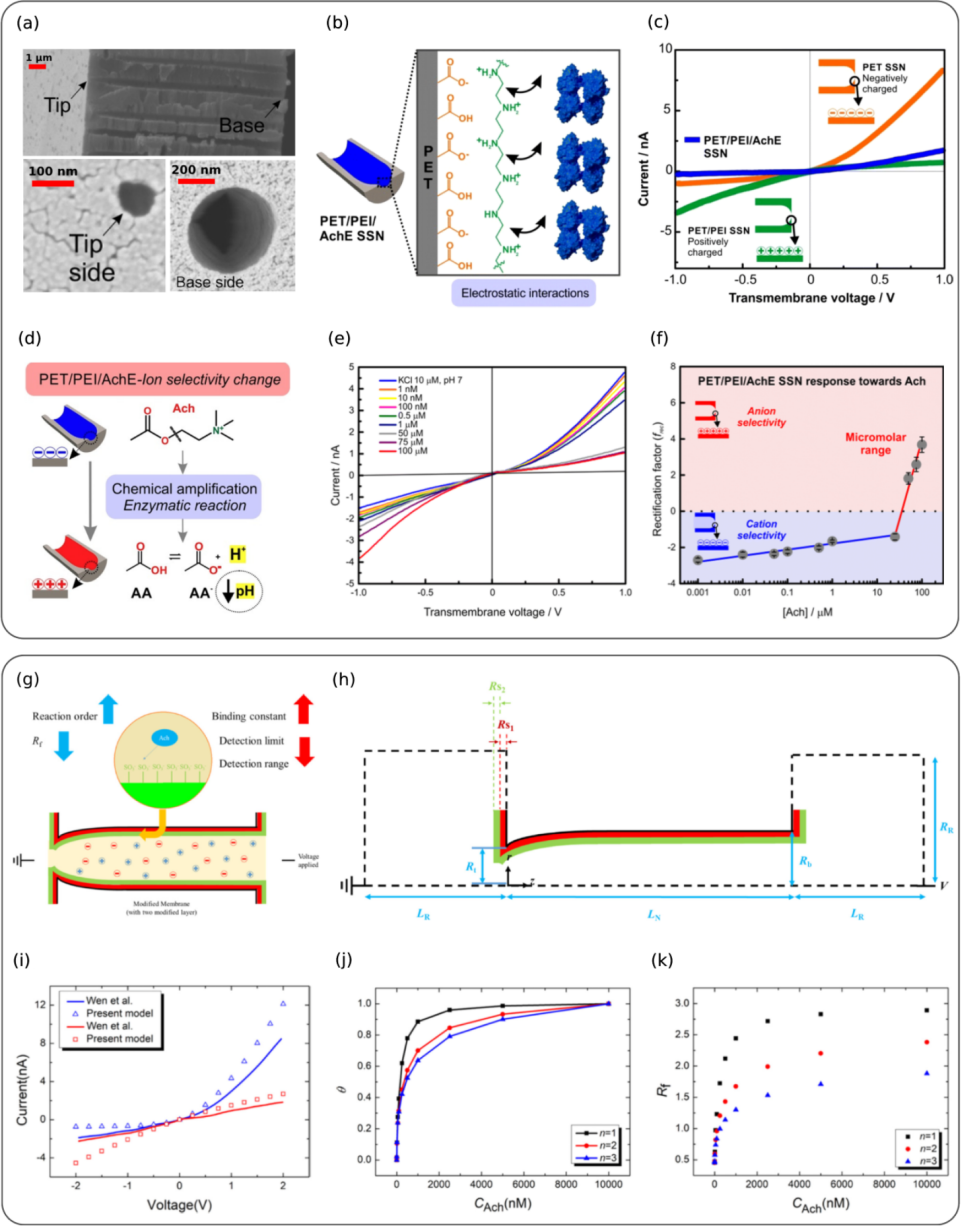
(a) SEM micrographs of PET SSN: base side (D ∼ 600 ± 28 nm), cross-section showing bullet shape, and tip side (d ∼ 85 ± 7 nm). (b) Schematic of electrostatic interactions between PET carboxylate groups, PEI amino groups, and AchE in PET/PET/AchE SSN. (c) I–V curves in 10 mM KCl (pH 7) for different modification steps: bare PET (orange), PEI-modified (green), and AchE-assembled (blue). (d) Surface charge changes upon Ach exposure. The enzymatic reaction produces acetic acid, lowering local pH and protonating – COO^–^ groups, making the surface charge more positive. (e) I–V curves at various Ach concentrations (0–100 μM) in 10 mM KCl (pH 7), recorded in situ after 30 min. (f) Frequency shift (frec) vs Ach concentration. Reprinted with permission from ref.^144^ Copyright 2022, Royal Society of Chemistry. (g) Modified membrane structure for Ach detection, showing binding constant, detection limit, and range. (h) Schematic of a bullet-shaped nanopore with tip radius R_t_, base radius R_b_, and length L_N_, connected to two cylindrical reservoirs. The nanopore surface has an inner PE layer (red, thickness R_s1_) and an outer PE layer (green, thickness R_s2_). The tip-side reservoir is grounded, and a voltage V is applied at the base-side. Dashed region denotes the computational domain. **(i)** I–V curves for PEI-modified (blue) and PEI+SCX4-modified (red) nanopores, comparing theoretical and experimental results. **(j)** Fractional surface coverage θ vs Ach concentration at pH 7 with K = 2 × 10^7^ L/mol for different n values. **(k)** Resistance factor R_f_ vs Ach concentration at pH 7 for K = 10^9^ L/mol. Reprinted with permission from ref.^141^ Copyright 2022, American Chemical Society.

In a related study, a synthetic bullet-shaped nanopore was functionalized at its tip with a dual-layer polyelectrolyte (PE) architecture comprising an inner layer of polyethyleneimine (PEI) and an outer layer of p-sulfonatocalix[4]­arene (SCX4).^144^ This specific surface modification strategy significantly enhanced ACh detection by improving signal resolution and reducing background noise, thereby facilitating the detection of trace-level ACh molecules (Figures 6g and 6h). As pre4sented in Table 3, the engineered nanopore exhibited optimized ion current rectification behavior, enabling highly sensitive monitoring of ACh interactions with the modified pore surface (Figure 6i-6k).

Despite these promising results, the broader implementation of this sensing approach faces several limitations. The platform’s performance is sensitive to variations in pH and ionic strength, which can compromise sensor stability and reproducibility. Furthermore, the detection mechanism, based on electrostatic interactions, introduces variability in the detection threshold and dynamic range, requiring rigorous calibration for reliable operation. The complexity of fabricating the dual-functionalized nanopore structure also presents challenges for scalability and integration into high-throughput or point-of-care diagnostic systems. Additionally, the use of nanopipette-based electrochemical interfaces for the simultaneous detection of ACh, tryptamine, and serotonin demonstrated the capability of a nanopore system to detect multiplex biomarkers by functionalized probe’s function, which monitors ionic transfer across a nanometer-scale liquid–liquid interface, allowing the selective detection of neurotransmitters based on their charge transfer characteristics.

### Nanopore-Based Detection of Histamine (Hm)

Histamine as a biogenic amine participates in various physiological processes, including neurotransmission, gastric acid secretion, and immune response. Abnormal levels of Hm are associated with allergic reactions, inflammatory diseases, and neurological disorders. The ability to detect Hm with high sensitivity and specificity is crucial for clinical diagnostics, food safety, and biomedical applications. Traditional detection techniques such as enzyme-linked immunosorbent assays (ELISA), chromatography, and electrochemical sensors often suffer from complex sample preparation, long analysis times, and interference from other biomolecules. Nanopore-based biosensors have emerged as a promising alternative due to their label-free detection, real-time analysis, and high sensitivity. In a related investigation, a nanofluidic sensor was developed for the label-free detection of Hm, utilizing a metal ion displacement strategy within asymmetric polymer nanopores functionalized with NTA-Ni^2+^ complexes.

This functionalization was achieved via covalent coupling of native carboxylic acid moieties with carboxylic acid groups with N,N-bis­(carboxymethyl)-l-lysine (BCML), as illustrated in Figure 7a. The sensor’s performance was evaluated through detailed electrical characterization (Figures 7b–c), which revealed a pronounced change in conductance correlated with varying Hm concentrations. The system exhibited notable specificity and sensitivity, successfully differentiating Hm from structurally similar neurotransmitters. These findings underscore the sensor’s promise for real-time monitoring of biologically relevant amines in complex environments (Figures 7b–f).^145^ In 2018, the Wolfgang Ensinger group introduced a biomolecular analyzer based on ion-conducting nanopores for the selective and label-free detection of histamine. This system operated by monitoring ionic current modulations resulting from histamine binding, thereby enabling precise molecular identification. The fabricated nanopores offer a robust and stable platform suitable for real-time biochemical sensing without the need for complex labeling protocols. The study advanced nanopore-based detection by employing functionalized polymer nanopores capable of high sensitivity and specificity in quantifying Hm under biological conditions (Table 3).

**Figure 7:**
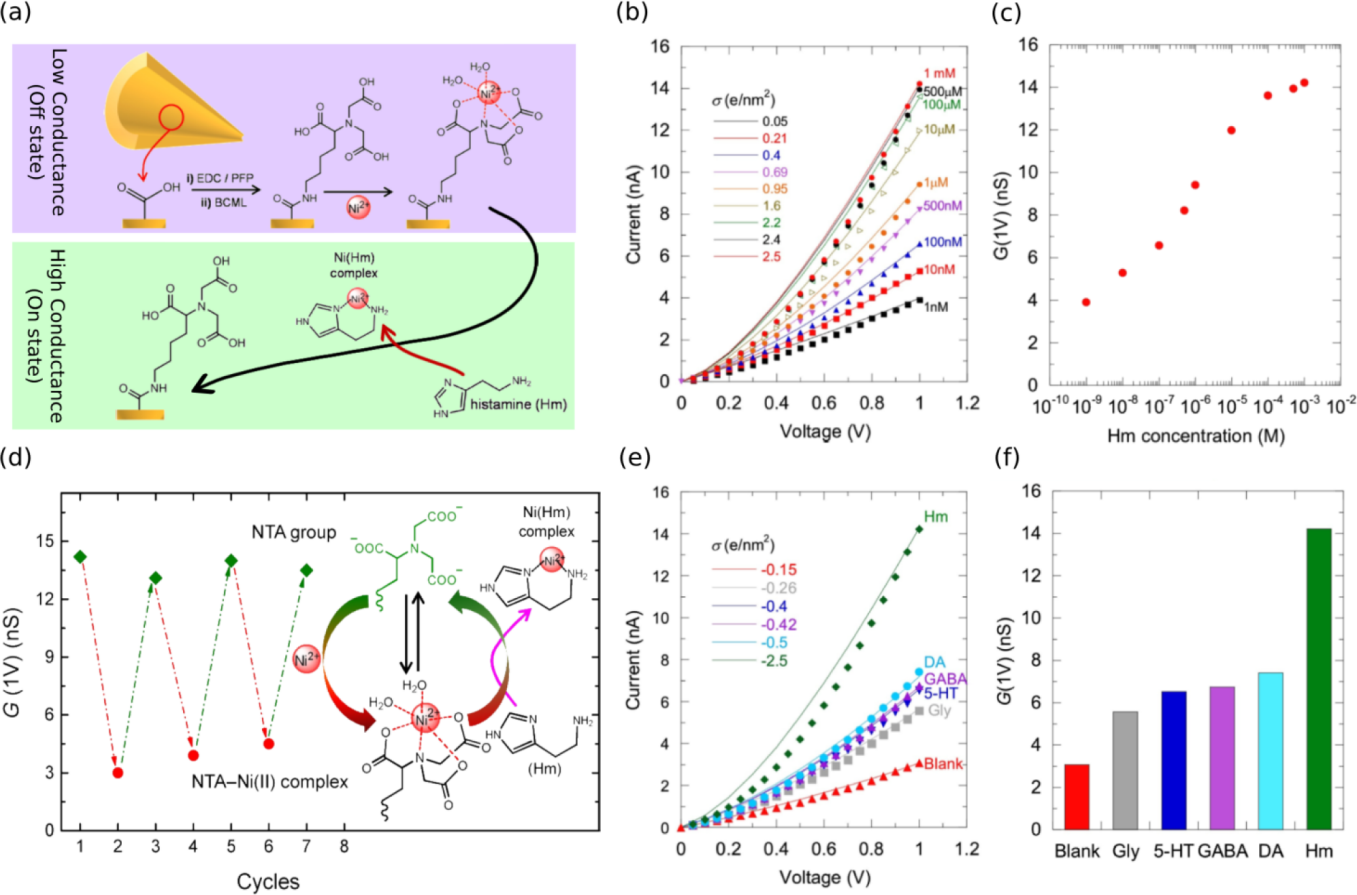
(a) Reaction scheme showing BCML chain functionalization with NTA, Ni­(II)–NTA complexation, and NTA regeneration by Ni^2+^ removal upon Hm exposure. (b) I–V curves for varying Hm concentrations. (c) Surface charge density vs Hm concentration, with sigmoid dependence indicating Ni-Hm equilibration and slower diffusion. (d) Conductance (G) cycles at 1 V, showing reversible Ni­(II) complexation/decomplexation. (e) I–V curves and (f) conductance changes of NTA-Ni^2+^ chelated pores (d = 45 nm, D = 800 nm) before and after exposure to 1 mM analytes: Gly, 5-HT, GABA, DA, and Hm. Reprinted with permission from ref.^145^ Copyright 2017, Elsevier.

## Challenges and Future Perspectives

Nanopore-based sensing technologies have emerged as powerful tools for the detection of neurotransmitters, offering single-molecule sensitivity, label-free detection, and potential for real-time monitoring. Despite these advantages, their broad application particularly in clinical and in vivo settings remains limited by several fundamental challenges. Chief among these is the difficulty in discriminating small-molecule neurotransmitters, which often exhibit similar physicochemical properties, such as size and charge. This similarity complicates differentiation using resistive pulse sensing systems, especially in complex biological environments where signal overlap is common.^172–174^ This limitation is further compounded by issues related to nanopore stability and reproducibility.^175–177^

Solid-state nanopores (SSNs), while offering notable advantages such as chemical robustness, thermal stability, and compatibility with integrated electronics, often suffer from high electrical noise and limited molecular selectivity. On the other hand, biological nanopores are typically more cost-effective and exhibit lower baseline noise, but they require delicate lipid membrane environments and controlled conditions that limit their practical utility. Recent advances in fabrication methods such as focused ion beam (FIB) milling, transmission electron microscopy (TEM), helium ion microscopy (HIM), and controlled breakdown (CBD) have enabled precise construction of sub-5 nm pores.^178^ However, ensuring their long-term operational integrity, especially in physiological fluids, remains an ongoing challenge.^179^

Closely tied to pore stability is the issue of signal fidelity. Rapid translocation of neurotransmitters through the nanopore often leads to brief, low-resolution events, making it difficult to extract meaningful data. Various strategies, including the use of electrophoretic and dielectrophoretic forces, have been proposed to slow down analyte passage and enhance signal-to-noise ratios.^180^ Techniques such as electrophoresis and dielectrophoretic can help regulate the passage of molecules through nanopores, enhancing detection accuracy. However, applied electric fields generate heat, further influencing SNR.^181^ Nevertheless, these approaches can introduce additional complexity, such as heat generation and increased system noise. Furthermore, low-frequency flicker noise, high-frequency dielectric interference, and limitations of the amplifier system itself continue to affect measurement precision. Optimizing the electrical readout while minimizing noise is therefore a central technical challenge for the field.^182^

To overcome these limitations, researchers have developed innovative device architectures aimed at improving both signal quality and analytical throughput. One notable advancement is the use of dual-barrel nanopipettes, which integrate a control and target sensing channel within a single probe.^183^ This configuration allows for differential measurement, enhances noise rejection, and enables simultaneous intracellular and extracellular monitoring of neurotransmitter dynamics. These systems not only improve temporal resolution but also allow multiplexed chemical sensing at the subcellular level an essential step toward more informative neurochemical mapping.

Equally important to signal clarity is molecular selectivity. To this end, functionalization of nanopores and nanopipettes with biorecognition elements such as aptamers, enzymes, or molecularly imprinted polymers has proven highly effective. Aptamers undergo conformational changes upon binding to their target neurotransmitters, producing distinct and detectable alterations in ionic current.^138^ This approach has successfully been used to differentiate neurotransmitters like dopamine in serum and artificial biofluids, offering high selectivity in otherwise noisy environments. However, environmental factors such as ionic strength and the presence of divalent cations can influence aptamer performance, highlighting the need for continued optimization of functional surface chemistries.^184^

Complementing these advances in sensor design are significant improvements in data analysis, particularly through the integration of artificial intelligence (AI) and machine learning (ML). These tools have shown remarkable potential in classifying transient current signatures, filtering noise, and even predicting signal drift. By training algorithms on large data sets of blockade events, researchers have achieved high accuracy in identifying specific neurotransmitters even those with overlapping current signatures.^185^ The ability to embed real-time AI-based processing into portable electronics suggests a promising future for autonomous, field-deployable nanopore sensors.^158^ These technical improvements collectively move the field closer to one of its most ambitious goals: in vivo neurotransmitter sensing. The small footprint and high spatial resolution of nanopipette-based sensors make them well-suited for implantation into brain tissue with minimal invasiveness. Early studies have demonstrated the potential for subcellular neurotransmitter detection at synaptic sites, suggesting that chronic monitoring of neurochemical activity in live animal models and eventually humans may soon be achievable. However, long-term stability, biocompatibility, and integration with minimally invasive recording systems remain areas of active research.

Beyond in vivo applications, nanopore sensors are being integrated into organ-on-a-chip and brain-on-a-chip systems for ex vivo analysis. These platforms enable real-time, spatially resolved monitoring of neurotransmitter release within engineered neural circuits, offering a powerful bridge between fundamental neuroscience and translational pharmacology. Such integration could transform drug screening pipelines by enabling multiplexed, high-throughput analysis of drug–neurotransmitter interactions in physiologically relevant.^186^ Simultaneously, the development of wearable and point-of-care devices is opening new avenues for continuous neurochemical monitoring in ambulatory settings. Flexible nanopipette arrays, combined with microfluidic interfaces and compact electronics, have shown preliminary success in tracking neurotransmitters such as dopamine in sweat or interstitial fluid. These systems hold promise for real-time mental health monitoring, noninvasive diagnostics, and personalized therapy management.^187^ Translating these prototypes into clinically viable devices, however, will require further work to ensure stability, reproducibility, and robust operation under variable physiological conditions.^188,189^

In the context of neurotechnology, nanopore platforms are poised to complement electrical brain-computer interfaces (BCIs) by enabling simultaneous decoding of chemical and electrophysiological signals. This convergence of chemical and electrical modalities could pave the way for next-generation BCIs that respond not only to action potentials but also to real-time fluctuations in neuromodulators such as dopamine and serotonin, thereby enhancing the fidelity of neural decoding and enabling new therapeutic paradigms in neuromodulation.^190,191^

Finally, to enhance nanopore-based neurotransmitter sensing, integrating state-of-the-art electronic measurement systems like the Axopatch amplifier (Axon Instruments Patch-Clamp Amplifiers) is essential. This amplifier offers the lowest noise and highest bandwidth available, making it the preferred choice for nanopore and nanopipette-based sensor systems. Its head-stage design allows for connection to the nanopore or nanopipette via long wires without compromising SNR. Additionally, the option to use external wires for measurements enhances user-friendliness and flexibility, making it suitable for a wide range of applications.^192^ Moreover, novel device architectures incorporating nanogap-based tunneling detectors, electronic transverse signal methods, and optical read-out technologies are being developed to further enhance detection capabilities.^125^ In this realm researchers are exploring ultrathin membranes, 2D materials, and zero-depth interfacial nanopores to enhance spatial resolution.^193^ Biosensor Nanotech Ltd., a leading company in nanopore technology, has developed advanced machinery to fabricate highly reliable nanopores, micropores, and nanofluidic systems.

## Conclusion

Nanopore-based sensing technology has demonstrated significant potential for the detection and quantification of neurotransmitters, offering high sensitivity, real-time monitoring, and label-free analysis. Solid-state nanopores (SSNs) and nanopipettes have shown promise in various applications, from single-cell analysis to the detection of neurotransmitters such as dopamine and acetylcholine. Despite the advancements, several challenges remain, including the need for improved sensitivity, stability, and reproducibility. The rapid translocation of analytes through nanopores and the complexity of nanopore fabrication are critical issues that need to be addressed. Future research should focus on refining nanopore fabrication techniques, enhancing molecular capture efficiency, and integrating advanced electronic measurement systems and machine learning algorithms to optimize performance. By overcoming these challenges, nanopore-based neurotransmitter detection can become a powerful tool for neurochemical research and clinical diagnostics, paving the way for early disease detection and personalized therapeutic interventions.

## Abbreviations

AD: Alzheimer’s disease
PD: Parkinson’s disease
ALS: Amyotrophic lateral sclerosis
CSF: Cerebrospinal fluid
HPLC: High-performance liquid chromatography
MS: Mass spectrometry
NMR: Nuclear magnetic resonance
ELISA: Enzyme-linked immunosorbent assay
FRET: Fluorescence resonance energy transfer
SSNs: Solid-state nanopores
Si_3_N_4_: Silicon nitride
TMDs: Transition metal dichalcogenides
FIB: Focused ion beam
FEB: Focused electron beam
EC: Electrochemical
MACE: Metal-assisted chemical etching
TEM: Transmission electron microscopy
SiNx: Silicon nitride
SiO_2_: Silicon oxide
Al_2_O_3_: Aluminum oxide
PET: Polyethylene terephthalate
RPS: Resistive pulse sensing
SSNs: Solid-state nanopores
AAO: Anodic aluminum oxide
CVD: Chemical vapor deposition
PVD: Physical vapor deposition
ALD: Atomic layer deposition
NLDA: Nanopore laser drilling algorithm
HIM: Helium ion microscopy
λd: Debye length
ICR: Ion current rectification
DA: Dopamine
SSN: Solid-state nanopore
ALD: Atomic layer deposition
ACh: Acetylcholine
LoD: Limit of detection
PET: Polyethylene terephthalate
PEI: Polyethylenimine
SCX4: *p*-Sulfonatocalix­[4]­arene
NTA–Ni2^+^: Nitrilotriacetic acid–nickel­(II)
GABA: Gamma-aminobutyric acid
SNR: Signal-to-noise ratio
TEM: Transmission electron microscopy
HIM: Helium ion microscopy
CBD: Controlled breakdown
ML: Machine learning
EC: Electrochemical
LSV: Linear sweep voltammetry
SPR: Surface plasmon resonance
SER: Surface-enhanced Raman spectroscopy
DPV: Differential pulse voltammetry
EG-FET: Electrolyte-gated field-effect transistor
UPLC-MS/MS: Ultraperformance liquid chromatography-tandem mass spectrometry
CA: Chronoamperometry
FET: Field effect transistor
MMOF: Metal organic frameworks
GQD: Graphene quantum dots
MIP: Molecular imprinted polymer
CNT: Carbon nanotubes
r-GO: Reduced graphene oxide
GNR: Graphene nanoribbons
COF: Covalent organic framework

## Vocabulary

Solid-state nanopore: A nanoscale pore fabricated in a solid membrane (e.g., SiN or graphene) used for single-molecule detection by monitoring ionic current changes during analyte translocation.
Nanopipette: A glass/quartz capillary pulled to a nanoscale tip, used for localized sensing, delivery, or ionic current measurements in nanofluidic and electrochemical applications.
Resistive pulse sensing: A technique that detects transient drops in ionic current as individual particles pass through a nanopore, providing size and concentration information.
Ion current rectification: Asymmetric current–voltage behavior in charged nanopores due to surface charge and geometry, often used for sensing and ion-selective gating.
Aptamer: A short, single-stranded DNA or RNA molecule that binds selectively to a target, used in biosensing due to its high affinity and specificity.
Debye length: The distance over which electrostatic interactions are screened in an electrolyte; defines the thickness of the electric double layer.

## References

[1] Shin K. C., Ali Moussa H. Y., Park Y. (2024). Cholesterol Imbalance and Neurotransmission
Defects in Neurodegeneration. Experimental &
Molecular Medicine.

[2] Benussi A., Premi E., Gazzina S., Cantoni V., Cotelli M. S., Giunta M., Gasparotti R., Calhoun V. D., Borroni B. (2020). Neurotransmitter
Imbalance Dysregulates Brain Dynamic Fluidity in Frontotemporal Degeneration. Neurobiol Aging.

[3] Nimgampalle M., Chakravarthy H., Sharma S., Shree S., Bhat A. R., Pradeepkiran J. A., Devanathan V. (2023). Neurotransmitter Systems in the Etiology
of Major Neurological Disorders: Emerging Insights and Therapeutic
Implications. Ageing Res. Rev..

[4] Cutler A. J., Mattingly G. W., Maletic V. (2023). Understanding the Mechanism of Action
and Clinical Effects of Neuroactive Steroids and GABAergic Compounds
in Major Depressive Disorder. Translational
Psychiatry.

[5] Hollestein V., Poelmans G., Forde N. J., Beckmann C. F., Ecker C., Mann C., Schäfer T., Moessnang C., Baumeister S., Banaschewski T., Bourgeron T., Loth E., Dell’Acqua F., Murphy D. G. M., Puts N. A., Tillmann J., Charman T., Jones E. J. H., Mason L., Ambrosino S., Holt R., Bölte S., Buitelaar J. K., Naaijen J. (2023). Excitatory/Inhibitory Imbalance in
Autism: The Role of Glutamate and GABA Gene-Sets in Symptoms and Cortical
Brain Structure. Translational Psychiatry.

[6] Wu T., Cai W., Chen X. (2023). Epigenetic Regulation of Neurotransmitter Signaling
in Neurological Disorders. Neurobiol Dis.

[7] Park S. A., Han S. M., Kim C. E. (2020). New Fluid
Biomarkers Tracking Non-Amyloid-β
and Non-Tau Pathology in Alzheimer’s Disease. Experimental & Molecular Medicine.

[8] Sangubotla R., Kim J. (2018). Recent Trends in Analytical
Approaches for Detecting Neurotransmitters
in Alzheimer’s Disease. TrAC Trends in
Analytical Chemistry.

[9] Chouliaras L., O’Brien J. T. (2023). The Use of Neuroimaging Techniques
in the Early and
Differential Diagnosis of Dementia. Molecular
Psychiatry.

[10] Nisar S., Haris M. (2023). Neuroimaging Genetics Approaches
to Identify New Biomarkers for the
Early Diagnosis of Autism Spectrum Disorder. Molecular Psychiatry.

[11] Chavan S. G., Rathod P. R., Koyappayil A., Hwang S., Lee M. H. (2025). Recent
Advances of Electrochemical and Optical Point-of-Care Biosensors for
Detecting Neurotransmitter Serotonin Biomarkers. Biosens Bioelectron.

[12] Nedelkov D., Nelson R. W. (2003). Surface Plasmon Resonance Mass Spectrometry:
Recent
Progress and Outlooks. Trends Biotechnol.

[13] Naik A. K., Hanay M. S., Hiebert W. K., Feng X. L., Roukes M. L. (2009). Towards
Single-Molecule Nanomechanical Mass Spectrometry. Nat. Nanotechnol..

[14] Rule, G. S. ; Hitchens, T. K. NMR Spectroscopy In Fundamentals of Protein NMR Spectroscopy Springer Science & Business Media 2006, 5 1–27 10.1007/1-4020-3500-4_1

[15] Jia C. P., Zhong X. Q., Hua B., Liu M. Y., Jing F. X., Lou X. H., Yao S. H., Xiang J. Q., Jin Q. H., Zhao J. L. (2009). Nano-ELISA for Highly
Sensitive Protein Detection. Biosens Bioelectron.

[16] Posch, A. , Ed. 2D PAGE: Sample Preparation and Fractionation; Methods in Molecular Biology; Humana Press: Totowa, NJ, 2008; Vol. 424. 10.1007/978-1-60327-064-9.

[17] Maveyraud L., Mourey L. (2020). Protein X-Ray Crystallography and
Drug Discovery. Molecules.

[18] Berlanda S. F., Breitfeld M., Dietsche C. L., Dittrich P. S. (2021). Recent Advances
in Microfluidic Technology for Bioanalysis and Diagnostics. Anal. Chem..

[19] Liu Y., Wang X., Campolo G., Teng X., Ying L., Edel J. B., Ivanov A. P. (2023). Single-Molecule Detection of α-Synuclein
Oligomers in Parkinson’s Disease Patients Using Nanopores. ACS Nano.

[20] He Y., Tsutsui M., Zhou Y., Miao X. S. (2021). Solid-State Nanopore
Systems: From Materials to Applications. NPG
Asia Materials.

[21] Xing X. L., Li W., Guo L. R., Wang K., Ma Y. Z., Zhao Q., Ji L. (2024). Nanopores in 2D Materials
and Their Applications in Single Molecule
Analysis. TrAC Trends Anal. Chem..

[22] Emilsson G., Sakiyama Y., Malekian B., Xiong K., Adali-Kaya Z., Lim R. Y. H., Dahlin A. B. (2018). Gating Protein Transport
in Solid
State Nanopores by Single Molecule Recognition. ACS Cent. Sci..

[23] Sze J. Y. Y., Ivanov A. P., Cass A. E. G., Edel J. B. (2017). Single
Molecule
Multiplexed Nanopore Protein Screening in Human Serum Using Aptamer
Modified DNA Carriers. Nat. Commun..

[24] Laucirica G., Toum Terrones Y., Cayón V., Cortez M. L., Toimil-Molares M. E., Trautmann C., Marmisollé W., Azzaroni O. (2021). Biomimetic Solid-State
Nanochannels for Chemical and Biological Sensing Applications. TrAC Trends in Analytical Chemistry.

[25] Stuber A., Douaki A., Hengsteler J., Buckingham D., Momotenko D., Garoli D., Nakatsuka N. (2023). Aptamer Conformational
Dynamics Modulate Neurotransmitter Sensing in Nanopores. ACS Nano.

[26] Vacca F., Galluzzi F., Blanco-Formoso M., Gianiorio T., De Fazio A. F., Tantussi F., Stürmer S., Haq W., Zrenner E., Chaffiol A., Joffrois C., Picaud S., Benfenati F., De Angelis F., Colombo E. (2024). Solid-State Nanopores
for Spatially Resolved Chemical Neuromodulation. Nano Lett..

[27] Duleba D., Johnson R. P. (2022). Sensing with Ion
Current Rectifying Solid-State Nanopores. Curr.
Opin Electrochem.

[28] Wang C., Sensale S., Pan Z., Senapati S., Chang H. C. (2021). Slowing
down DNA Translocation through Solid-State Nanopores by Edge-Field
Leakage. Nat. Commun..

[29] Zhou Y., Long X., Zhang Y., Zheng D., Jiang Y., Hu Y. (2025). Advances and Challenges
in Solid-State Nanopores for DNA Sequencing. Langmuir.

[30] Liu H., Zhou Q., Wang W., Fang F., Zhang J. (2023). Solid-State
Nanopore Array: Manufacturing and Applications. Small.

[31] Liu R., Liu Z., Li J., Qiu Y. (2024). Low-Cost and Convenient Fabrication
of Polymer Micro/Nanopores with the Needle Punching Process and Their
Applications in Nanofluidic Sensing. Biomicrofluidics.

[32] Chen Y., Chen Y., Long J., Shi D., Chen X., Hou M., Gao J., Liu H., He Y., Fan B., Wong C. P., Zhao N. (2021). Achieving a Sub-10 Nm Nanopore Array
in Silicon by Metal-Assisted Chemical Etching and Machine Learning. International Journal of Extreme Manufacturing.

[33] Zvuloni E., Zrehen A., Gilboa T., Meller A. (2021). Fast and Deterministic
Fabrication of Sub-5 Nanometer Solid-State Pores by Feedback-Controlled
Laser Processing. ACS Nano.

[34] Macha M., Marion S., Tripathi M., Thakur M., Lihter M., Kis A., Smolyanitsky A., Radenovic A. (2022). High-Throughput Nanopore Fabrication
and Classification Using Xe-Ion Irradiation and Automated Pore-Edge
Analysis. ACS Nano.

[35] White R. J., Ervin E. N., Yang T., Chen X., Daniel S., Cremer P. S., White H. S. (2007). Single Ion-Channel
Recordings Using
Glass Nanopore Membranes. J. Am. Chem. Soc..

[36] Shi W., Friedman A. K., Baker L. A. (2017). Nanopore
Sensing. Anal. Chem..

[37] Wang H., Tang H., Qiu X., Li Y. (2024). Solid-State Glass Nanopipettes:
Functionalization and Applications. *Chemistry – A*. European Journal.

[38] Kececi K., Dinler A., Kaya D. (2022). Review—Nanopipette
Applications
as Sensors, Electrodes, and Probes: A Study on Recent Developments. J. Electrochem. Soc..

[39] Lin K., Chen C., Wang C., Lian P., Wang Y., Xue S., Sha J., Chen Y. (2022). Fabrication of Solid-State Nanopores. Nanotechnology.

[40] Jeong C., Jung J., Sheppard K., Choi C. H. (2023). Control of the Nanopore
Architecture of Anodic Alumina via Stepwise Anodization with Voltage
Modulation and Pore Widening. Nanomaterials.

[41] Cao L., Wang Y. (2009). Fabrication and Investigation
of Single Track-Etched Nanopore and
Its Applications. Radiat. Meas..

[42] Zhang Y., Miyahara Y., Derriche N., Yang W., Yazda K., Capaldi X., Liu Z., Grutter P., Reisner W. (2019). Nanopore Formation
via Tip-Controlled Local Breakdown Using an Atomic Force Microscope. Small Methods.

[43] Willmott G. R., Vogel R., Yu S. S. C., Groenewegen L. G., Roberts G. S., Kozak D., Anderson W., Trau M. (2010). Use of Tunable
Nanopore Blockade Rates to Investigate Colloidal Dispersions. J. Phys.: Condens. Matter.

[44] Shen Y., Zhang Z., Fukuda T. (2015). Bending Spring
Rate Investigation
of Nanopipette for Cell Injection. Nanotechnology.

[45] Bell N. A. W., Engst C. R., Ablay M., Divitini G., Ducati C., Liedl T., Keyser U. F. (2012). DNA Origami
Nanopores. Nano Lett..

[46] Yuan Z., Lin Y., Hu J., Wang C. (2024). Controllable Fabrication of Sub-10
Nm Graphene Nanopores via Helium Ion Microscopy and DNA Detection. Biosensors.

[47] Jia R., Mirkin M. V. (2020). The Double Life of Conductive Nanopipette: A Nanopore
and an Electrochemical Nanosensor. Chem. Sci..

[48] Albrecht T. (2019). Single-Molecule
Analysis with Solid-State Nanopores. Annu. Rev.
Anal. Chem..

[49] Varongchayakul N., Song J., Meller A., Grinstaff M. W. (2018). Single-Molecule
Protein Sensing in a Nanopore: A Tutorial. Chem.
Soc. Rev..

[50] Yang L., Yamamoto T. (2016). Quantification of Virus Particles Using Nanopore-Based
Resistive-Pulse Sensing Techniques. Front. Microbiol..

[51] Yilmaz D., Kaya D., Kececi K., Dinler A. (2021). Role of Nanopore
Geometry
in Particle Resolution by Resistive-Pulse Sensing. ChemistrySelect.

[52] Miles B. N., Ivanov A. P., Wilson K. A., Dogan F., Japrung D., Edel J. B. (2013). Single Molecule
Sensing with Solid-State Nanopores:
Novel Materials, Methods, and Applications. Chem. Soc. Rev..

[53] Lu Y. T., Barron A. R. (2014). Anti-Reflection Layers Fabricated by a One-Step Copper-Assisted
Chemical Etching with Inverted Pyramidal Structures Intermediate between
Texturing and Nanopore-Type Black Silicon. J.
Mater. Chem. A Mater..

[54] Ying Y. L., Hu Z. L., Zhang S., Qing Y., Fragasso A., Maglia G., Meller A., Bayley H., Dekker C., Long Y. T. (2022). Nanopore-Based Technologies
beyond DNA Sequencing. Nat. Nanotechnol..

[55] Kowalczyk S. W., Grosberg A. Y., Rabin Y., Dekker C. (2011). Modeling the Conductance
and DNA Blockade of Solid-State Nanopores. Nanotechnology.

[56] Ma L., Li Z., Yuan Z., Huang C., Siwy Z. S., Qiu Y. (2020). Modulation
of Ionic Current Rectification in Ultrashort Conical Nanopores. Anal. Chem..

[57] Grover N. B., Naaman J., Ben-Sasson S., Doljanski F. (1972). Electrical
Sizing of Particles in Suspensions. 3. Rigid Spheroids and Red Blood
Cells. Biophys. J..

[58] Steinbock L. J., Krishnan S., Bulushev R. D., Borgeaud S., Blokesch M., Feletti L., Radenovic A. (2014). Probing the
Size of Proteins with
Glass Nanopores. Nanoscale.

[59] DeBlois R. W., Bean C. P. (1970). Counting and Sizing
of Submicron Particles by the Resistive
Pulse Technique. Rev. Sci. Instrum..

[60] Ledden B., Fologea D., Talaga D. S., Li J., Iqbal S. M., Bashir R. (2011). Sensing Single Protein Molecules
with Solid-State Nanopores. Nanopores.

[61] Armstrong J. K., Wenby R. B., Meiselman H. J., Fisher T. C. (2004). The Hydrodynamic
Radii of Macromolecules and Their Effect on Red Blood Cell Aggregation. Biophys. J..

[62] Carlsen A., Tabard-Cossa V. (2022). Mapping Shifts
in Nanopore Signal to Changes in Protein
and Protein-DNA Conformation. Proteomics.

[63] Yusko E. C., Bruhn B. R., Eggenberger O. M., Houghtaling J., Rollings R. C., Walsh N. C., Nandivada S., Pindrus M., Hall A. R., Sept D., Li J., Kalonia D. S., Mayer M. (2016). Real-Time Shape Approximation and
Fingerprinting of Single Proteins Using a Nanopore. Nat. Nanotechnol..

[64] Gouy M. (1910). Sur La Constitution
de La Charge Électrique à La Surface d’un Électrolyte. Journal de Physique Théorique et Appliquée.

[65] Chau C., Actis P., Hewitt E. (2020). Methods for Protein Delivery into
Cells: From Current Approaches to Future Perspectives. Biochem. Soc. Trans..

[66] Tsujino K., Matsumura M. (2005). Boring Deep
Cylindrical Nanoholes in Silicon Using
Silver Nanoparticles as a Catalyst. Adv. Mater..

[67] Zheng H., Han M., Zheng P., Zheng L., Qin H., Deng L. (2014). Porous Silicon
Templates Prepared by Cu-Assisted Chemical Etching. Mater. Lett..

[68] Van
Toan N., Inomata N., Toda M., Ono T. (2018). Ion Transport by Gating
Voltage to Nanopores Produced via Metal-Assisted Chemical Etching
Method. Nanotechnology.

[69] De
Ferrari F., Raja S. N., Herland A., Niklaus F., Stemme G. (2025). Sub-5 Nm Silicon Nanopore Sensors: Scalable Fabrication
via Self-Limiting Metal-Assisted Chemical Etching. ACS Appl. Mater. Interfaces.

[70] Bruera F. A., Kramer G. R., Vera M. L., Ares A. E. (2019). Synthesis and Morphological
Characterization of Nanoporous Aluminum Oxide Films by Using a Single
Anodization Step. Coatings.

[71] Beri R., Kushwaha M. K., Grover N. (2017). A Review on
Studies of Mechanical
Properties of Anodized Alumina Oxide. Int. J.
Res. Eng. Technol..

[72] Masuda H., Fukuda K. (1995). Ordered Metal Nanohole Arrays Made by a Two-Step Replication
of Honeycomb Structures of Anodic Alumina. Science
(1979).

[73] Mínguez-Bacho I., Scheler F., Büttner P., Bley K., Vogel N., Bachmann J. (2018). Ordered Nanopore Arrays
with Large Interpore Distances
via One-Step Anodization. Nanoscale.

[74] Yang J., Pan T., Liu T., Mao C., Ho H. P., Yuan W. (2025). Angular-Inertia
Regulated Stable and Nanoscale Sensing of Single Molecules Using Nanopore-In-A-Tube. Adv. Mater..

[75] Lanzavecchia G., Kuttruff J., Doricchi A., Douaki A., Kumaranchira
Ramankutty K., García I., Lin L., Viejo Rodríguez A., Wågberg T., Krahne R., Maccaferri N., Garoli D. (2023). Plasmonic Photochemistry as a Tool to Prepare Metallic
Nanopores with Controlled Diameter for Optimized Detection of Single
Entities. Adv. Opt Mater..

[76] Ayub M., Ivanov A., Hong J., Kuhn P., Instuli E., Edel J. B., Albrecht T. (2010). Precise Electrochemical
Fabrication
of Sub-20 Nm Solid-State Nanopores for Single-Moleculebiosensing. J. Phys.: Condens. Matter.

[77] Chen Z., Zhang H. (2005). Mechanisms for Formation
of a One-Dimensional Horizontal Anodic Aluminum
Oxide Nanopore Array on a Si Substrate. J. Electrochem.
Soc..

[78] Ateş S., Baran E., Yazıcı B. (2018). The Nanoporous
Anodic Alumina Oxide
Formed by Two-Step Anodization. Thin Solid Films.

[79] Hartwig S., Klages C.-P. (2014). Ordered 2D Nanopores
in Nickel Electrodeposits–A
Self-Organization Phenomenon. J. Electrochem.
Soc..

[80] Hui F., Li B., He P., Hu J., Fang Y. (2009). Electrochemical Fabrication
of Nanoporous Polypyrrole Film on HOPG Using Nanobubbles as Templates. Electrochem commun.

[81] Elias J., Parlinska-Wojtan M., Erni R., Niederberger C., Sauvage F., Thevenin M., Michler J., Philippe L. (2012). Passing the
Limit of Electrodeposition: ‘Gas Template’ H2 Nanobubbles
for Growing Highly Crystalline Nanoporous ZnO. Nano Energy.

[82] Tarábková H., Janda P. (2016). Single-Step Nanoporation
of Water-Immersed Polystyrene Film by Gaseous
Nanobubbles. Langmuir.

[83] Yuan Z., Lei X., Wang C. (2020). Controllable
Fabrication of Solid State Nanopores Array
by Electron Beam Shrinking. Int. J. Mach Tools
Manuf.

[84] Li J., Fan C., Ding J., Xue S., Chen Y., Li Q., Wang H., Zhang X. (2017). In Situ Heavy
Ion Irradiation Studies
of Nanopore Shrinkage and Enhanced Radiation Tolerance of Nanoporous
Au. Sci. Rep..

[85] Li J., Stein D., McMullan C., Branton D., Aziz M. J., Golovchenko J. A. (2001). Ion-Beam
Sculpting at Nanometre Length Scales. Nature.

[86] Spende A., Sobel N., Lukas M., Zierold R., Riedl J. C., Gura L., Schubert I., Moreno J. M. M., Nielsch K., Stühn B., Hess C., Trautmann C., Toimil-Molares M. E. (2015). TiO2, SiO2,
and Al2O3 Coated Nanopores and Nanotubes
Produced by ALD in Etched Ion-Track Membranes for Transport Measurements. Nanotechnology.

[87] Wang Y., Deng T., Chen Q., Liang F., Liu Z. (2016). Highly Efficient
Shrinkage of Inverted-Pyramid Silicon Nanopores by Plasma-Enhanced
Chemical Vapor Deposition Technology. Nanotechnology.

[88] Chen P., Mitsui T., Farmer D. B., Golovchenko J., Gordon R. G., Branton D. (2004). Atomic Layer Deposition to Fine-Tune
the Surface Properties and Diameters of Fabricated Nanopores. Nano Lett..

[89] Asghar W., Ilyas A., Billo J. A., Iqbal S. M. (2011). Shrinking of Solid-State
Nanopores by Direct Thermal Heating. Nanoscale
Res. Lett..

[90] Gilboa T., Zvuloni E., Zrehen A., Squires A. H., Meller A. (2020). Automated,
Ultra-Fast Laser-Drilling of Nanometer Scale Pores and Nanopore Arrays
in Aqueous Solutions. Adv. Funct Mater..

[91] Li W., Bell N. A. W., Hernández-Ainsa S., Thacker V. V., Thackray A. M., Bujdoso R., Keyser U. F. (2013). Single Protein Molecule
Detection by Glass Nanopores. ACS Nano.

[92] Leva C. V., Jain S., Kistermann K., Sakurai K., Stemme G., Herland A., Mayer J., Niklaus F., Raja S. N. (2025). Localized
Nanopore Fabrication in Silicon Nitride Membranes by Femtosecond Laser
Exposure and Subsequent Controlled Breakdown. ACS Appl. Mater. Interfaces.

[93] Park S. R., Peng H., Ling X. S. (2007). Fabrication of Nanopores in Silicon
Chips Using Feedback Chemical Etching. Small.

[94] Deng T., Li M., Chen J., Wang Y., Liu Z. (2014). Controllable Fabrication
of Pyramidal Silicon Nanopore Arrays and Nanoslits for Nanostencil
Lithography. J. Phys. Chem. C.

[95] Chen Q., Wang Y., Deng T., Liu Z. (2018). Fabrication of Nanopores
and Nanoslits with Feature Sizes down to 5 Nm by Wet Etching Method. Nanotechnology.

[96] Dutt S., Karawdeniya B. I., Bandara Y. M. N. D. Y., Afrin N., Kluth P. (2023). Ultrathin,
High-Lifetime Silicon Nitride Membranes for Nanopore Sensing. Anal. Chem..

[97] Tseng A. A. (2005). Recent
Developments in Nanofabrication Using Focused Ion Beams. Small.

[98] Russo C. J., Golovchenko J. A. (2012). Atom-by-Atom Nucleation and Growth of Graphene Nanopores. Proc. Natl. Acad. Sci. U. S. A..

[99] Morin A., Lucot D., Ouerghi A., Patriarche G., Bourhis E., Madouri A., Ulysse C., Pelta J., Auvray L., Jede R., Bruchhaus L., Gierak J. (2012). FIB Carving of Nanopores into Suspended Graphene Films. Microelectron. Eng..

[100] Biance A. L., Gierak J., Bourhis É., Madouri A., Lafosse X., Patriarche G., Oukhaled G., Ulysse C., Galas J. C., Chen Y., Auvray L. (2006). Focused Ion Beam Sculpted
Membranes for Nanoscience Tooling. Microelectron.
Eng..

[101] Wu M.-Y., Krapf D., Zandbergen M., Zandbergen H., Batson P. E. (2005). Formation of Nanopores in a SiN/SiO_2_ Membrane with an Electron Beam. Appl.
Phys. Lett..

[102] Kim J. Y., Han D., Crouch G. M., Kwon S. R., Bohn P. W. (2019). Capture of Single Silver Nanoparticles
in Nanopore
Arrays Detected by Simultaneous Amperometry and Surface-Enhanced Raman
Scattering. Anal. Chem..

[103] Fürjes P. (2019). Controlled Focused Ion Beam Milling
of Composite Solid
State Nanopore Arrays for Molecule Sensing. Micromachines.

[104] Lo C. J., Aref T., Bezryadin A. (2006). Fabrication
of Symmetric Sub-5 Nm Nanopores Using Focused Ion and Electronbeams. Nanotechnology.

[105] Wanunu M., Sutin J., McNally B., Chow A., Meller A. (2008). DNA Translocation Governed by Interactions with Solid-State
Nanopores. Biophys. J..

[106] Kuan A. T., Golovchenko J. A. (2012). Nanometer-Thin
Solid-State Nanopores
by Cold Ion Beam Sculpting. Appl. Phys. Lett..

[107] Niedzwiecki D. J., Iyer R., Borer P. N., Movileanu L. (2013). Sampling a
Biomarker of the Human Immunodeficiency Virus across a Synthetic Nanopore. ACS Nano.

[108] Liu S., Lu B., Zhao Q., Li J., Gao T., Chen Y., Zhang Y., Liu Z., Fan Z., Yang F., You L., Yu D. (2013). Boron Nitride Nanopores:
Highly Sensitive DNA Single-Molecule Detectors. Adv. Mater..

[109] Larkin J., Henley R. Y., Muthukumar M., Rosenstein J. K., Wanunu M. (2014). High-Bandwidth Protein Analysis Using
Solid-State Nanopores. Biophys. J..

[110] Akahori R., Haga T., Hatano T., Yanagi I., Ohura T., Hamamura H., Iwasaki T., Yokoi T., Anazawa T. (2014). Slowing Single-Stranded DNA Translocation
through a
Solid-State Nanopore by Decreasing the Nanopore Diameter. Nanotechnology.

[111] Lee M. H., Kumar A., Park K. B., Cho S. Y., Kim H. M., Lim M. C., Kim Y. R., Kim K. B. (2014). A Low-Noise
Solid-State Nanopore Platform Based on a Highly Insulating Substrate. Sci. Rep..

[112] Feng Y., Zhang Y., Ying C., Wang D., Du C. (2015). Nanopore-Based
Fourth-Generation DNA Sequencing Technology. Genomics Proteomics Bioinformatics.

[113] Md Ibrahim N. N. N., Hashim A. M. (2021). High Sensitivity
of Deoxyribonucleic
Acid Detection via Graphene Nanohole/Silicon Micro-Nanopore Structure
Fabricated by Focused Ion Beam. Mater. Lett..

[114] Malekian B., Xiong K., Kang E. S. H., Andersson J., Emilsson G., Rommel M., Sannomiya T., Jonsson M. P., Dahlin A. (2019). Optical Properties of Plasmonic Nanopore
Arrays Prepared by Electron Beam and Colloidal Lithography. Nanoscale Adv..

[115] Kim M. J., McNally B., Murata K., Meller A. (2007). Characteristics
of Solid-State Nanometre Pores Fabricated Using a Transmission Electronmicroscope. Nanotechnology.

[116] Wanunu M., Dadosh T., Ray V., Jin J., McReynolds L., Drndić M. (2010). Rapid Electronic Detection of Probe-Specific
MicroRNAs Using Thin Nanopore Sensors. Nat.
Nanotechnol..

[117] Wanunu M., Bhattacharya S., Xie Y., Tor Y., Aksimentiev A., Drndic M. (2011). Nanopore Analysis of Individual RNA/Antibiotic
Complexes. ACS Nano.

[118] Zhang H., Zhao Q., Tang Z., Liu S., Li Q., Fan Z., Yang F., You L., Li X., Zhang J., Yu D. (2013). Slowing Down DNA Translocation Through
Solid-State Nanopores by Pressure. Small.

[119] Traversi F., Raillon C., Benameur S. M., Liu K., Khlybov S., Tosun M., Krasnozhon D., Kis A., Radenovic A. (2013). Detecting the Translocation of DNA through a Nanopore
Using Graphene Nanoribbons. Nat. Nanotechnol..

[120] Venta K., Shemer G., Puster M., Rodríguez-Manzo J. A., Balan A., Rosenstein J. K., Shepard K., Drndić M. (2013). Differentiation
of Short, Single-Stranded DNA Homopolymers in Solid-State Nanopores. ACS Nano.

[121] Henley R. Y., Ashcroft B. A., Farrell I., Cooperman B. S., Lindsay S. M., Wanunu M. (2016). Electrophoretic Deformation of Individual
Transfer RNA Molecules Reveals Their Identity. Nano Lett..

[122] Experton J., Wu X., Martin C. R. (2017). From Ion
Current
to Electroosmotic Flow Rectification in Asymmetric Nanopore Membranes. Nanomaterials.

[123] Siwy Z., Heins E., Harrell C. C., Kohli P., Martin C. R. (2004). Conical-Nanotube Ion-Current Rectifiers: The Role of
Surface Charge. J. Am. Chem. Soc..

[124] White R. J., Zhang B., Daniel S., Tang J. M., Ervin E. N., Cremer P. S., White H. S. (2006). Ionic Conductivity
of the Aqueous Layer Separating a Lipid Bilayer Membrane and a Glass
Support. Langmuir.

[125] Heins E. A., Siwy Z. S., Baker L. A., Martin C. R. (2005). Detecting
Single Porphyrin Molecules in a Conically Shaped Synthetic Nanopore. Nano Lett..

[126] Chen L., He H., Jin Y. (2015). Counting and Dynamic
Studies of the Small Unilamellar Phospholipid Vesicle Translocation
with Single Conical Glass Nanopores. Anal. Chem..

[127] Terejánszky P., Makra I., Fürjes P., Gyurcsányi R. E. (2014). Calibration-Less Sizing and Quantitation of Polymeric
Nanoparticles and Viruses with Quartz Nanopipets. Anal. Chem..

[128] Wang Y., Kececi K., Mirkin M. V., Mani V., Sardesai N., Rusling J. F. (2013). Resistive-Pulse
Measurements with
Nanopipettes: Detection of Au Nanoparticles and Nanoparticle-Bound
Anti-Peanut IgY. Chem. Sci..

[129] Lin X., Ivanov A. P., Edel J. B. (2017). Selective
Single Molecule Nanopore
Sensing of Proteins Using DNA Aptamer-Functionalised Gold Nanoparticles. Chem. Sci..

[130] Wen C., Dematties D., Zhang S. L. (2021). A Guide to Signal Processing Algorithms
for Nanopore Sensors. ACS Sens..

[131] Kawaguchi T., Tsutsui M., Murayama S., Leong I. W., Yokota K., Komoto Y., Taniguchi M. (2024). Enhanced Nanoparticle
Sensing in a Highly Viscous Nanopore. Small
Methods.

[132] Zhang S., Chen W., Song L., Wang X., Sun W., Song P., Ashraf G., Liu B., Zhao Y. Di. (2021). Recent
Advances in Ionic Current Rectification Based Nanopore Sensing: A
Mini-Review. Sensors and Actuators Reports.

[133] Abraham D. A., Li A. D., Sanmugam A., Wadaan M. A., Baabbad A., Kanagaraj K., Karuppasamy K., Maiyalagan T., Kim H. S., Vikraman D. (2024). Highly Sensitive
and
Selective Detection of Dopamine Using Atomic Layer Deposited HfO2
Ultra-Thin Films. Electrochim. Acta.

[134] Zhao T., Wang J. W., Zhang H. S., Zheng X., Chen Y. P., Tang H., Jiang J. H. (2022). Development
of Dual-Nanopore
Biosensors for Detection of Intracellular Dopamine and Dopamine Efflux
from Single PC12 Cell. Anal. Chem..

[135] Colombo M. L., McNeil S., Iwai N., Chang A., Shen M. (2016). Electrochemical Detection of Dopamine
via Assisted Ion Transfer at
Nanopipet Electrode Using Cyclic Voltammetry. J. Electrochem. Soc..

[136] Yang D., Liu G., Li H., Liu A., Guo J., Shan Y., Wang Z., He J. (2020). The Fabrication of
a Gold Nanoelectrode–Nanopore Nanopipette for Dopamine Enrichment
and Multimode Detection. Analyst.

[137] Yang C., Hu K., Wang D., Zubi Y., Lee S. T., Puthongkham P., Mirkin M. V., Venton B. J. (2019). Cavity
Carbon-Nanopipette Electrodes for Dopamine Detection. Anal. Chem..

[138] Burdina, A. Translating Aptamer-Modified Nanopipettes to Complex Systems; Master’s Thesis, Politecnico di Torino: Turin, Italy, 2022 https://webthesis.biblio.polito.it/24791/.

[139] Ma Y., Ma Y., Liu K., Wang D., Liu R., Chen Q., Jiang D., Pan R. (2024). An Ultra-Sensitive
Platinized Nanocavity Electrode for Analysis of Cytosolic Catecholamines
in One Living Cell. Talanta.

[140] He C., Tao M., Zhang C., He Y., Xu W., Liu Y., Zhu W. (2022). Microelectrode-Based
Electrochemical Sensing Technology
for in Vivo Detection of Dopamine: Recent Developments and Future
Prospects. Crit. Rev. Anal. Chem..

[141] Chung C. Y., Hsu J. P. (2022). Nanosensing of Acetylcholine
Molecules:
Influence of the Association Mechanism. Langmuir.

[142] Kuanaeva R. M., Vaneev A. N., Gorelkin P. V., Erofeev A. S. (2024). Nanopipettes
as a Potential Diagnostic Tool for Selective Nanopore Detection of
Biomolecules. Biosensors (Basel).

[143] Wei J., Hong H., Wang X., Lei X., Ye M., Liu Z. (2024). Nanopore-Based Sensors for DNA Sequencing:
A Review. Nanoscale.

[144] Toum Terrones Y., Laucirica G., Cayón V. M., Fenoy G. E., Cortez M. L., Toimil-Molares M. E., Trautmann C., Mamisollé W. A., Azzaroni O. (2022). Highly Sensitive Acetylcholine
Biosensing via Chemical Amplification of Enzymatic Processes in Nanochannels. Chem. Commun..

[145] Ali M., Ramirez P., Duznovic I., Nasir S., Mafe S., Ensinger W. (2017). Label-Free Histamine
Detection with Nanofluidic Diodes
through Metal Ion Displacement Mechanism. Colloids
Surf. B Biointerfaces.

[146] Shou M., Ferrario C. R., Schultz K. N., Robinson T. E., Kennedy R. T. (2006). Monitoring Dopamine in Vivo by Microdialysis
Sampling
and On-Line CE-Laser-Induced Fluorescence. Anal.
Chem..

[147] Uutela P., Karhu L., Piepponen P., Käenmäki M., Ketola R. A., Kostiainen R. (2009). Discovery
of Dopamine Glucuronide in Rat and Mouse Brain Microdialysis Samples
Using Liquid Chromatography Tandem Mass Spectrometry. Anal. Chem..

[148] Kumar S., Thakur M., Kumari S., Sharma S., Kanwar S. S. (2025). Chromium-Functionalized
Metal-Organic Frameworks as
Highly Sensitive, Dual-Mode Sensors for Real Time and Rapid Detection
of Dopamine. Talanta.

[149] Liu H., Li N., Zhang H., Zhang F., Su X. (2018). A Simple and
Convenient Fluorescent Strategy for the Highly Sensitive Detection
of Dopamine and Ascorbic Acid Based on Graphene Quantum Dots. Talanta.

[150] He W., Gui R., Jin H., Wang B., Bu X., Fu Y. (2018). Ratiometric Fluorescence and Visual Imaging Detection
of Dopamine
Based on Carbon Dots/Copper Nanoclusters Dual-Emitting Nanohybrids. Talanta.

[151] Sharma R., Jaryal V. B., Sharma P., Rana D. S., Sheel A., Fouad D., Gupta N., Singh D. (2025). Fabrication
of Carbon-Based Electrochemical Sensor Derived from Waste Coconut
Husk for Dopamine Detection in Human Urine. J. Electrochem. Soc..

[152] Jaryal V. B., Kumar S., Singh D., Gupta N. (2024). Thiourea-Modified
Multiwalled Carbon Nanotubes as Electrochemical Biosensor for Ultra-Precise
Detection of Dopamine. ChemNanoMat.

[153] Sun C. L., Lee H. H., Yang J. M., Wu C. C. (2011). The Simultaneous
Electrochemical Detection of Ascorbic Acid, Dopamine, and Uric Acid
Using Graphene/Size-Selected Pt Nanocomposites. Biosens Bioelectron.

[154] Florescu M., David M. (2017). Tyrosinase-Based Biosensors
for Selective
Dopamine Detection. Sensors.

[155] Sharma S., Gupta B. D. (2018). Surface Plasmon
Resonance Based Highly
Selective Fiber Optic Dopamine Sensor Fabricated Using Molecular Imprinted
GNP/SnO2 Nanocomposite. Journal of Lightwave
Technology.

[156] Tang L., Li S., Han F., Liu L., Xu L., Ma W., Kuang H., Li A., Wang L., Xu C. (2015). SERS-Active Au@Ag Nanorod Dimers for Ultrasensitive Dopamine Detection. Biosens Bioelectron.

[157] Singh D., Srivastava A., Chaturvedi V. K., Singh J. (2025). Nanostructured WS2@Chitosan-Modified Screen-Printed Carbon Electrodes
for Efficient Amperometric Detection of Histamine. ACS Omega.

[158] Putri F. R., Munir M. A., Rahmawati F., Gunawan A. (2025). Polyurethane-Electrode Modified to Determine Histamine
in Selected Samples Using Chemical Sensor. AIP
Conf. Proc..

[159] Basozabal I., Guerreiro A., Gomez-Caballero A., Aranzazu Goicolea M., Barrio R. J. (2014). Direct Potentiometric
Quantification
of Histamine Using Solid-Phase Imprinted Nanoparticles as Recognition
Elements. Biosens Bioelectron.

[160] Wang W., Feng R., Wei K., Xu J., Dong W., Li J., Sun J., Wang S., Mao X. (2025). An Integrated Colorimetric Biosensing Platform Containing Microneedle
Patches and Aptasensor for Histamine Monitoring in Seafood. J. Hazard Mater..

[161] Shkodra B., Petrelli M., Yang K. A., Tagliaferri A., Lugli P., Petti L., Nakatsuka N. (2024). Polymeric
Integration of Structure-Switching Aptamers on Transistors for Histamine
Sensing. Faraday Discuss..

[162] Tran Q. H., Nguyen T. T., Pham K. P. (2020). Development
of the
High Sensitivity and Selectivity Method for the Determination of Histamine
in Fish and Fish Sauce from Vietnam by UPLC-MS/MS. Int. J. Anal. Chem..

[163] De Bundel D., Sarre S., Van Eeckhaut A., Smolders I., Michotte Y. (2008). Critical Evaluation of Acetylcholine
Determination in Rat Brain Microdialysates Using Ion-Pair Liquid Chromatography
with Amperometric Detection. Sensors.

[164] Yamamoto K., Sato K., Chikuma T., Kato T. (2004). A Highly Sensitive
and Stable Detection of Acetylcholine by HPLC-Osmium-Horseradish Peroxidase
Redox Polymer Electrode Coated on a Gold Radial Flow Ring Disk. Anal. Chim. Acta.

[165] Sattarahmady N., Heli H., Vais R. D. (2013). An Electrochemical
Acetylcholine Sensor Based on Lichen-like Nickel Oxide Nanostructure. Biosens Bioelectron.

[166] Pitiphattharabun S., Auewattanapun K., Htet T. L., Thu M. M., Panomsuwan G., Techapiesancharoenkij R., Ohta J., Jongprateep O. (2024). Reduced Graphene
Oxide/Zinc Oxide Composite as an Electrochemical
Sensor for Acetylcholine Detection. Sci. Rep..

[167] McClain E. S., Miller D. R., Cliffel D. E. (2019). Communication—Microfluidic
Electrochemical Acetylcholine Detection in the Presence of Chlorpyrifos. J. Electrochem. Soc..

[168] Zhang Y., Ding L., Xiao B., Wang S., Meng W., Gao L., Che T., Zheng X. (2025). Ti3C2MXene/GNRs
for Synergistically Highly Enhanced Sensitivity of Optical Fiber SPR
Acetylcholine Biosensors via an Electrostatic Layer-by-Layer Assembly
Method. Biosens Bioelectron.

[169] Fu C., Li Y., Lei X., Su J., Chen Y., Wu Y., Shi W., Tan X., Li Y., Jung Y. M. (2024). SERS Sensor
for Acetylcholine Detection Based on Covalent Organic Framework Hybridized
Gold Nanoparticles As Nanozymes. Anal. Chem..

[170] Guo J., Wu S., Wang Y., Zhao M. (2020). A Label-Free Fluorescence
Biosensor Based on a Bifunctional MIL-101­(Fe) Nanozyme for Sensitive
Detection of Choline and Acetylcholine at Nanomolar Level. Sens Actuators B Chem..

[171] Chae M. S., Yoo Y. K., Kim J., Kim T. G., Hwang K. S. (2018). Graphene-Based Enzyme-Modified Field-Effect Transistor
Biosensor for Monitoring Drug Effects in Alzheimer’s Disease
Treatment. Sens Actuators B Chem..

[172] Tang Z., Zhang D., Cui W., Zhang H., Pang W., Duan X. (2016). Fabrications, Applications
and Challenges
of Solid-State Nanopores: A Mini Review. Nanomater.
Nanotechnol..

[173] Salehirozveh M., Bonné R., Kumar P., Abazar F., Dehghani P., Mijakovic I., Roy V. A. L. (2025). Enhanced Detection
of Brain-Derived Neurotrophic Factor (BDNF) Using a Reduced Graphene
Oxide Field-Effect Transistor Aptasensor. Nanoscale.

[174] Dehghani P., Karthikeyan V., Tajabadi A., Assi D. S., Catchpole A., Wadsworth J., Leung H. Y., Roy V. A. L. (2024). Rapid
Near-Patient Impedimetric Sensing Platform for Prostate Cancer Diagnosis. ACS Omega.

[175] Gong X., Patil A. V., Ivanov A. P., Kong Q., Gibb T., Dogan F., Demello A. J., Edel J. B. (2014). Label-Free
in-Flow Detection of Single DNA Molecules Using Glass Nanopipettes. Anal. Chem..

[176] Salehirozveh M., Kure Larsen A., Stojmenovic M., Thei F., Dong M. (2023). In-Situ PLL-g-PEG
Functionalized
Nanopore for Enhancing Protein Characterization. Chem. Asian J..

[177] Salehirozveh M., Porro A., Thei F. (2023). Large-Scale Production
of Polyimide Micropore-Based Flow Cells for Detecting Nano-Sized Particles
in Fluids. RSC Adv..

[178] Lanza M., Gao T., Yin Z., Zhang Y., Liu Z., Tong Y., Shen Z., Duan H. (2013). Nanogap Based Graphene
Coated AFM Tips with High Spatial Resolution. Conductivity and Durability. Nanoscale.

[179] Dehghani P., Salehirozveh M., Tajabadi A., Yeung C. C., Lam M., Leung H. Y., Roy V. A. L. (2025). Next-Gen Point-of-Care Tool for Ultra-Sensitive
Detection of Urinary Spermine for Prostate Cancer Diagnosis. ACS Sens..

[180] Tao C., Bai Y., Chen J., Lu J., Bi Y., Li J. (2024). Detection of Glutamate Decarboxylase
Antibodies and Simultaneous
Multi-Molecular Translocation Exploration by Glass Nanopores. Biosensors.

[181] Guo W., Cao L., Xia J., Nie F. Q., Wen M., Xue J., Song Y., Zhu D., Wang Y., Jiang L. (2010). Energy Harvesting
with Single-Ion-Selective Nanopores: A Concentration-Gradient-Driven
Nanofluidic Power Source. Adv. Funct. Mater..

[182] Coasne B., Gubbins K. E., Pellenq R. J. M. (2005). Temperature
Effect
on Adsorption/Desorption Isotherms for a Simple Fluid Confined within
Various Nanopores. Adsorption.

[183] Stuber A., Cavaccini A., Manole A., Burdina A., Massoud Y., Patriarchi T., Karayannis T., Nakatsuka N. (2024). Interfacing Aptamer-Modified Nanopipettes
with Neuronal
Media and Ex Vivo Brain Tissue. ACS Meas. Sci.
Au.

[184] Fan R., Karnik R., Yue M., Li D., Majumdar A., Yang P. (2005). DMA Translocation in Inorganic Nanotubes. Nano
Lett..

[185] Komoto Y., Ohshiro T., Yoshida T., Tarusawa E., Yagi T., Washio T., Taniguchi M. (2020). Time-Resolved
Neurotransmitter Detection in Mouse Brain Tissue Using an Artificial
Intelligence-Nanogap. Sci. Rep..

[186] Yu Z., Tang D. (2022). Artificial Neural Network-Assisted
Wearable Flexible
Sweat Patch for Drug Management in Parkinson’s Patients Based
on Vacancy-Engineered Processing of g-C3N4. Anal. Chem..

[187] Zhang J., Cheng Z., Li P., Tian B. (2025). Materials
and Device Strategies to Enhance Spatiotemporal Resolution in Bioelectronics. Nat. Rev. Mater..

[188] Rubby M. F., Fonder C., Uchayash S., Liang X., Sakaguchi D. S., Que L. (2024). Assessment of the Behaviors
of an
In Vitro Brain Model On-Chip under Shockwave Impacts. ACS Appl. Mater. Interfaces.

[189] Jayant K., Wenzel M., Bando Y., Hamm J. P., Mandriota N., Rabinowitz J. H., Plante I. J.-L., Owen J. S., Sahin O., Shepard K. L., Yuste R. (2019). Flexible Nanopipettes
for Minimally Invasive Intracellular Electrophysiology In Vivo. Cell Rep..

[190] Meller A., Nivon L., Brandin E., Golovchenko J., Branton D. (2000). Rapid Nanopore Discrimination between Single Polynucleotide
Molecules. Proc. Natl. Acad. Sci. U. S. A..

[191] Salehirozveh M., Dehghani P., Zimmermann M., Roy V. A. L., Heidari H. (2020). Graphene Field Effect Transistor
Biosensors Based on Aptamer for Amyloid-β Detection. IEEE Sens J..

[192] Dzubiella J., Hansen J. P. (2005). Electric-Field-Controlled
Water and
Ion Permeation of a Hydrophobic Nanopore. J.
Chem. Phys..

[193] Freedman K. J., Otto L. M., Ivanov A. P., Barik A., Oh S. H., Edel J. B. (2016). Nanopore Sensing
at Ultra-Low Concentrations
Using Single-Molecule Dielectrophoretic Trapping. Nat. Commun..

